# Evaluating the performance of tools used to call minority variants from whole genome short-read data

**DOI:** 10.12688/wellcomeopenres.13538.2

**Published:** 2018-09-13

**Authors:** Khadija Said Mohammed, Nelson Kibinge, Pjotr Prins, Charles N. Agoti, Matthew Cotten, D.J. Nokes, Samuel Brand, George Githinji

**Affiliations:** 1 Pwani University, Kilifi, Kenya; 2 KEMRI-Wellcome Trust Research Programme, KEMRI Centre for Geographic Medicine Research – Coast, Kilifi, Kenya; 3 University Medical Center Utrecht, Utrecht, The Netherlands; 4 Virosciences Department, Erasmus Medical Centre, Rotterdam, The Netherlands; 5 School of Life Sciences and Zeeman Institute (SBIDER), University of Warwick, Coventry, UK

**Keywords:** variant calling, minority variants, concordance, performance, RSV

## Abstract

**Background: **High-throughput whole genome sequencing facilitates investigation of minority virus sub-populations from virus positive samples. Minority variants are useful in understanding within and between host diversity, population dynamics and can potentially assist in elucidating person-person transmission pathways. Several minority variant callers have been developed to describe low frequency sub-populations from whole genome sequence data. These callers differ based on bioinformatics and statistical methods used to discriminate sequencing errors from low-frequency variants.

**Methods: **We evaluated the diagnostic performance and concordance between published minority variant callers used in identifying minority variants from whole-genome sequence data from virus samples. We used the ART-Illumina read simulation tool to generate three artificial short-read datasets of varying coverage and error profiles from an RSV reference genome. The datasets were spiked with nucleotide variants at predetermined positions and frequencies. Variants were called using FreeBayes, LoFreq, Vardict, and VarScan2. The variant callers’ agreement in identifying known variants was quantified using two measures; concordance accuracy and the inter-caller concordance.

**Results: **The variant callers reported differences in identifying minority variants from the datasets. Concordance accuracy and inter-caller concordance were positively correlated with sample coverage. FreeBayes identified the majority of variants although it was characterised by variable sensitivity and precision in addition to a high false positive rate relative to the other minority variant callers and which varied with sample coverage. LoFreq was the most conservative caller.

**Conclusions: **We conducted a performance and concordance evaluation of four minority variant calling tools used to identify and quantify low frequency variants. Inconsistency in the quality of sequenced samples impacts on sensitivity and accuracy of minority variant callers. Our study suggests that combining at least three tools when identifying minority variants is useful in filtering errors when calling low frequency variants.

## Introduction

RNA viruses have been described as a population of closely related sequences that arise from rapid genomic evolution coupled with a high replication and mutation rates ( Domingo *et al.*, 2012; Eigen *et al.*, 1988; Holland *et al.*, 1992). Genetic changes in RNA viruses result from genetic drift, erroneous replication processes, mutagenic agents and upon which natural selection acts ( Moya *et al.*, 2004). Rapid replication and mutations generate an ensemble of mutant genomes that are comprised of both dominant and low frequency variants. This diversity has been shown to affect virus fitness landscape, transmission, colonization and replication ( Henn *et al.*, 2012; Stack *et al.*, 2013; Vignuzzi *et al.*, 2006).

Many recent studies ( Henn *et al.*, 2012; Poon *et al.*, 2016; Stack *et al.*, 2013) have demonstrated the potential application of virus diversity to inform person-to-person transmission during virus outbreaks. A number of methods that incorporate both genomic and epidemiologic data to infer pathogen transmission have recently been developed ( Worby *et al.,* 2017). These approaches rely partly on the accurate detection and quantification of minority variant populations from genomic samples.

Several tools have been developed to identify and quantify minority variants from short-read data ( Koboldt *et al.*, 2009; Koboldt *et al.*, 2012; Lai *et al.*, 2016; Macalalad *et al.*, 2012; Wilm *et al.*, 2012; Yang *et al.*, 2013). Nonetheless, these tools do not fully account for discrepancies that arise from sample collection, pre-processing and sequencing in addition to errors that are introduced during downstream bioinformatic analysis. Rigorous quality control in sample processing and analysis is often suggested to distinguish true biological variants from artefactual variants ( Zhang & Flaherty, 2017). In some cases, sequencing errors can be reduced by developing high-fidelity protocols and laboratory quality control measures ( Kinde *et al.*, 2011; McCrone & Lauring, 2016; Watson *et al.*, 2013). Additionally, the uncertainty resulting from random sequencing errors can be countered by sequencing larger populations at higher coverage ( Zukurov *et al.*, 2016). A number of studies have extensively explored variants from somatic or tumour samples ( Hofmann *et al.*, 2017; Koboldt *et al.*, 2012; Krøigård *et al.*, 2016; Lai *et al.*, 2016; Pabinger *et al.*, 2014) and their application in clinical genomics, but only a limited number of studies have explored the nature of variants from patient-derived samples that target viral populations ( Henn *et al.*, 2012; Macalalad *et al.*, 2012; Wilm *et al.*, 2012; Yang *et al.*, 2013; Zukurov *et al.*, 2016) and especially when calling variants from respiratory viruses such as the respiratory syncytial virus (RSV).

In this study, we evaluated four published minority variant detection tools using artificial short-read data with different error profiles. We explored the tools’ ability to detect and quantify minority variants and assessed their overall agreement which we defined using two metrics, concordance accuracy, which measures the combined accuracy of the variant callers, and inter-caller concordance, which is the size of the largest set of variant callers that agree at each position. We show that concordance metrics are dependent on sample coverage and are influenced by the quality of input data.

## Methods

**Figure 1.**
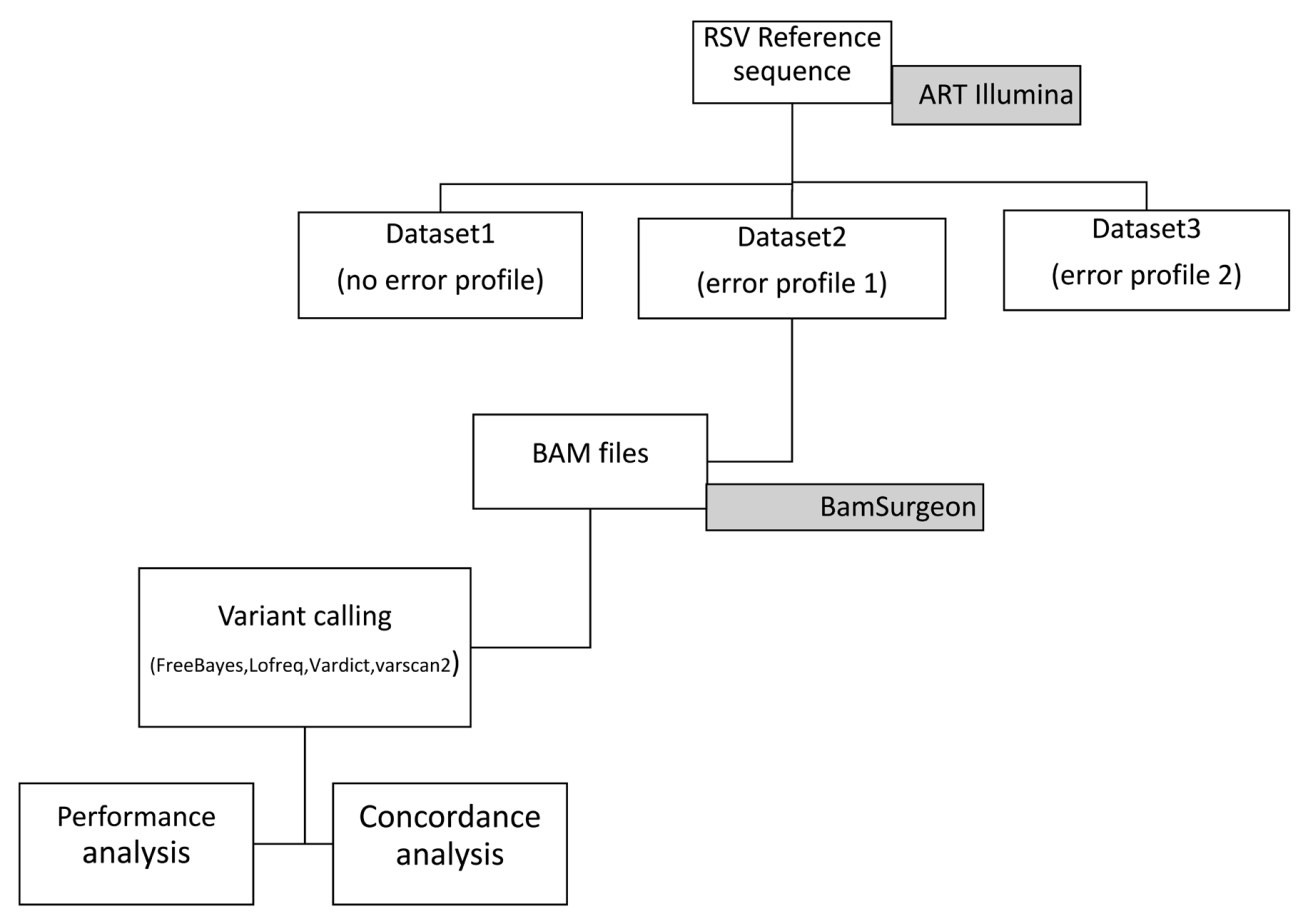
A schematic diagram showing the variant calling workflow. The artificial datasets (BAM files) were generated using ART-Illumina based on an RSV reference genome. BAMSurgeon was used to spike the resulting BAM files by inserting known variants at known locations across the artificial BAM file.

Overall, we considered ten published, open-source tools with presumed ability to call minority variants from virus deep sequence data. A number of callers were excluded from the analysis for various reasons, for example, the GATK HaplotypeCaller primarily targets germline calling from human and not variant calling from viral samples. We experienced technical difficulties in setting up the Platypus caller and even after setup, Platypus did not provide calls across all levels of coverage in our datasets. SAMtools mpileup did not provide direct allele frequencies while V-Phaser was superseded by V-Phaser 2 which has reported bugs and could not handle reads aligned with BWA-MEM. Therefore, the following four tools were evaluated, FreeBayes version 1.1.0-3-g961e5f3, LoFreq version 2.1.2, VarDict version 30.3.17 and VarScan version 2.4.2. A schematic diagram showing the overall approach is shown in Figure 1.

### Artificial datasets

Artificial datasets were generated based on an RSV reference sequence (GenBank accession number KX510245.1) using ART-Illumina version 2.5.8 ( Huang *et al.*, 2012). ART-Illumina was took the reference RSV genome sequence as input and generated artificial reads using data derived error models to mimic sequence data. Each dataset comprised of eight samples with varying depth of coverage (20, 50, 100, 500, 1000, 2000, 5000, 10000) and was generated using the methods described in Supplementary File 1, section S1.1. The first dataset did not incorporate an error profile. Error profile models (empirical error models based on the distribution of base quality scores) were generated from uncompressed FastQC raw reads derived from sequenced RSV whole genome samples, referred to as “good” and a “bad” sample based on FastQC metrics ( Supplementary File 2 and Supplementary File 3) and used to generate artificial reads in dataset 2 and 3. ART-Illumina-generated artificial SAM files were converted to the BAM format, sorted and indexed using SAMtools version 1.3.1 for each dataset.

166 randomly and uniformly generated nucleotide mutations at different frequencies ( Supplementary Table 1) were inserted into each of the artificial datasets using BamSurgeon ( Ewing *et al.*, 2015), such that a base change was made amongst the reads at each alignment position as described in Supplementary File 1, section S1.2. This process was repeated with a separate set of 155 positions that comprised a set of mutations with frequencies below 0.5 ( Supplementary Table 1).

### Variant calling

The BAM files from each of the three datasets were used as input to each of the four variant callers (FreeBayes, Lofreq, VarDict and VarScan2). The default parameter options used in each tool are explicitly provided in Supplementary File 4. All output files were provided in the variant call format (VCF) or as a tabular file for the case of VarDict. The output from the VCF and tabular file was parsed and written as a comma separated (CSV) file.

### Performance measures

To evaluate the performance of the variant calling algorithms, we compared the sequence generated by each variant caller *vc*, denoted ${\text{S}}^{\text{vc}}={\left({\text{S}}_{\text{i}}^{\text{vc}}\in \{\text{A},\text{C},\text{T},\text{G}\}\right)}_{\text{i}=1,...,\text{N}},$ to the gold standard "spiked" sequence, denoted *S ^true^*, at each of *N=*15205 nucleotide positions. The accuracy of each variant caller is the normalized Hamming distance from the gold standard sequence, $\frac{1}{N}\sum_{i=1}^{N}d(S_{i}^{true},S_{i}^{v}),$ where *d*(x,y) is the standard discrete metric giving 1 when x=y, and 0 otherwise. By distinguishing between the sets of positions where variants did and did not occur in the gold standard sequence we calculated sensitivity, specificity, precision and accuracy ( Table 1).

**Table 1.** A breakdown of performance metrics of variant callers evaluated from first dataset that did not incorporate an error profile. The samples represent simulated datasets of varying depth of coverage. True positive (TP), true negative (TN), false positive (FP) and false negatives (FN) were used to calculate performance metrics of each caller. FPR – False positive rate.

Sample	Caller	TP	TN	FP	FN	Sensitivity	Specificity	Precision	FPR	Accuracy
**1 (20X)**	freebayes	87	15018	21	79	0.5241	0.9986	0.8056	0.0014	0.9934
	lofreq	29	15039	0	137	0.1747	1	1	0	0.991
	vardict	72	15039	0	94	0.4337	1	1	0	0.9938
	varscan	11	15039	0	155	0.0663	1	1	0	0.9898
**2 (50X)**	freebayes	118	14901	138	47	0.7152	0.9908	0.4609	0.00918	0.9878
	lofreq	67	15039	0	99	0.40361	1	1	0	0.9935
	vardict	108	15039	0	58	0.6506	1	1	0	0.9962
	varscan	22	15039	0	144	0.13253	1	1	0	0.9905
**3 (100X)**	freebayes	127	14454	585	38	0.7697	0.9611	0.1784	0.0389	0.959
	lofreq	57	15039	0	109	0.3434	1	1	0	0.9928
	vardict	104	15038	1	62	0.6265	0.9999	0.9905	6.65E-05	0.9959
	varscan	40	15039	0	126	0.241	1	1	0	0.9917
**4 (500X)**	freebayes	131	12559	2480	30	0.8137	0.8351	0.0502	0.1649	0.8349
	lofreq	60	15039	0	106	0.3614	1	1	0	0.993
	vardict	110	15029	10	56	0.6627	0.9993	0.9167	6.65E-04	0.9957
	varscan	73	15039	0	93	0.4398	1	1	0	0.9939
**5 (1000X)**	freebayes	146	14414	625	20	0.8795	0.9584	0.1894	0.0416	0.9576
	lofreq	57	15039	0	109	0.3434	1	1	0	0.9928
	vardict	109	15036	3	57	0.6567	0.9998	0.9732	1.99E-04	0.9961
	varscan	79	15039	0	87	0.4759	1	1	0	0.9943
**6 (2000X)**	freebayes	146	14923	116	20	0.8795	0.9923	0.5571	0.0077	0.9911
	lofreq	70	15039	0	96	0.4217	1	1	0	0.9937
	vardict	120	15039	0	46	0.7229	1	1	0	0.997
	varscan	83	15039	0	83	0.5	1	1	0	0.9945
**7 (5000X)**	freebayes	149	15020	19	17	0.8976	0.9987	0.8869	0.0013	0.9976
	lofreq	67	15039	0	99	0.40366	1	1	0	0.9935
	vardict	117	15036	3	49	0.7048	0.9998	0.975	1.99E-04	0.9966
	varscan	78	15039	0	88	0.4699	1	1	0	0.9942
**8 (10000X)**	freebayes	145	15022	17	21	0.8735	0.9989	0.8951	0.0011	0.9975
	lofreq	72	15039	0	94	0.4337	1	1	0	0.9938
	vardict	118	15038	1	48	0.7108	0.9999	0.9916	6.65E-05	0.9968
	varscan	97	15039	0	69	0.5843	1	1	0	0.9955

### Concordance analysis

We defined two concordance metrics to present the level of agreement between different callers in detecting the same variant positions in the sequence. The first concordance metric is concordance accuracy, which measures the combined accuracy of the variant callers. At the true variant position *i* we then $C_{\mathrm{acc}}(i)=\sum_{\mathrm{vc}=1}^{4}d(S_{i}^{\mathrm{true}},S_{i}^{vc}),$ which can be either 0, 1, 2, 3, 4 for each true variant position. The second concordance metric is inter-caller concordance, which is the size of the largest set of variant callers that agree at each position *i*, without reference to any gold standard sequence. We used both bar plots and heat maps to visualize the effect of coverage on C _*acc*_. Visualization of inter-caller concordance for variant sets was achieved using a bar plot and expounded by UpSet plots ( Lex *et al.*, 2014) in R version 3.4.2.

## Results

**Figure 2.**
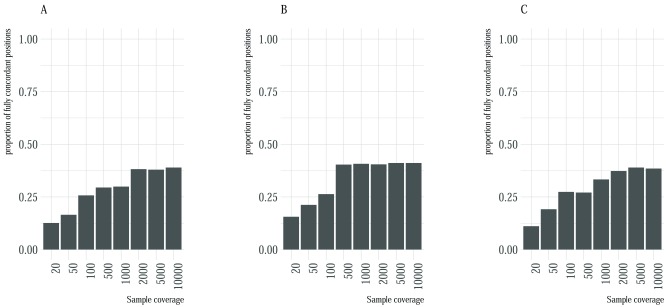
Proportion of fully concordant positions with respect to sample coverage. Each plot **A**– **C** represents the proportion (y-axis) of fully concordant variants with respect to read coverage (x-axis) for the first, second and third dataset. Concordant positions were defined as positions that were identified by all the four variant callers.

We used three artificial datasets of varying coverage and error profile to assess the concordance accuracy and inter-caller concordance for four minority variant callers. The first dataset comprised of artificial reads based on an RSV genome, the second dataset comprised of the similar simulated set of reads whilst incorporating an error profile from the set of reads used to assemble the reference genome, the third dataset was generated using an error profile from a poorly sequenced sample. Overall, concordance accuracy improved with increase in sample coverage ( Figure 2), and the proportion of positions that could not be identified by any variant caller decreased with increase in coverage ( Supplementary Figure 1). For all the three datasets, and at each coverage level, fully concordant variants were below 50% of the total variants suggesting that considering only fully concordant positions eliminated a substantial number of variant positions. There were marginal improvements in the number of concordant variants in the second dataset compared to the first and the third error profile. Across all datasets, there was little improvement at detecting fully concordant positions after a coverage of 2000 ( Figure 2). We utilized UpSetR plots to provide a visual summary of the combination of variant callers that contributed to the observed concordance accuracy (Supplementary Figure 4).

**Figure 3.**
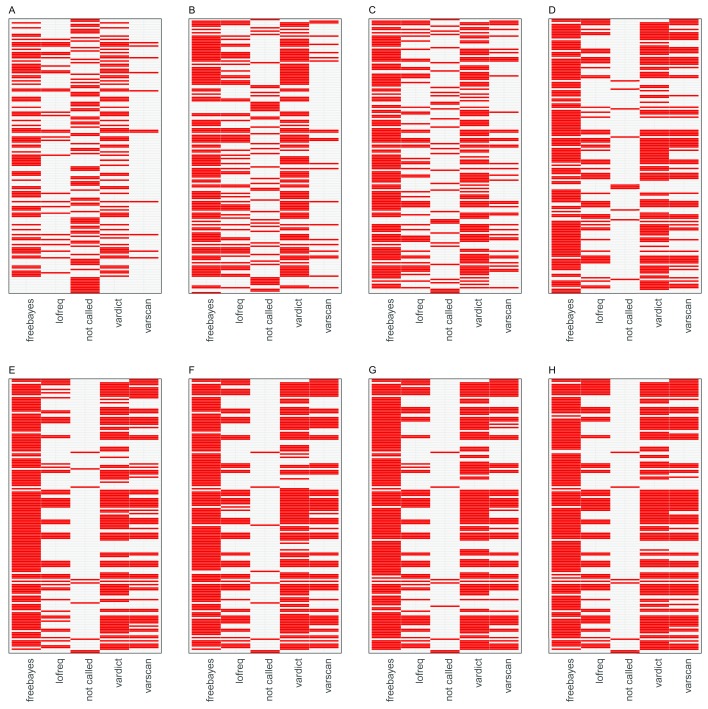
Heat maps illustrating tool specific concordance for the first artificial dataset. The red tiles represent variants detected by each caller from the list of 166 variant positions. The panels are arranged left to right **A**– **H** in the order of increasing sample coverage (20,50,100,500,1000,2000,5000 and 10,000). The “not called” column in each panel represents the variants that were not identified by any of the variant callers.

**Figure 4.**
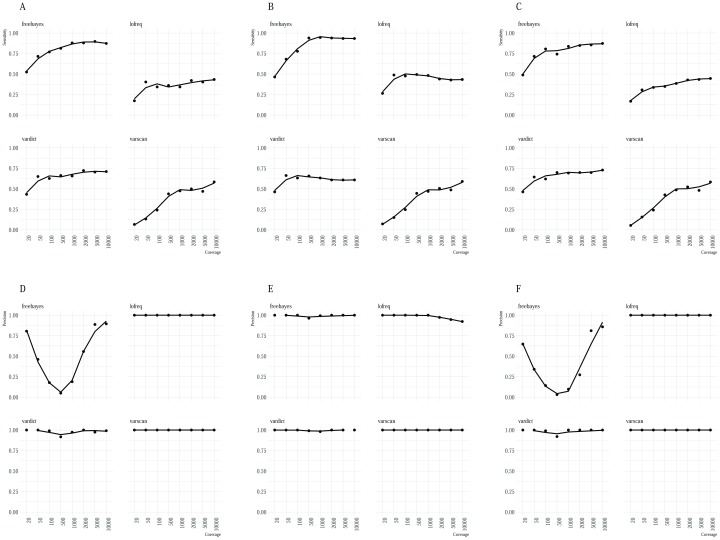
A summary of the relationship between sample coverage, sensitivity ( **A**– **C**) and sample coverage and precision ( **D**– **F**). The x-axis shows the sample coverage and the y-axis represents the sensitivity and precision respectively. Sensitivity of the callers rose gradually from low to high coverage samples. Again, precision was variable for FreeBayes calls while relatively high for the rest of the three callers.

**Figure 5.**
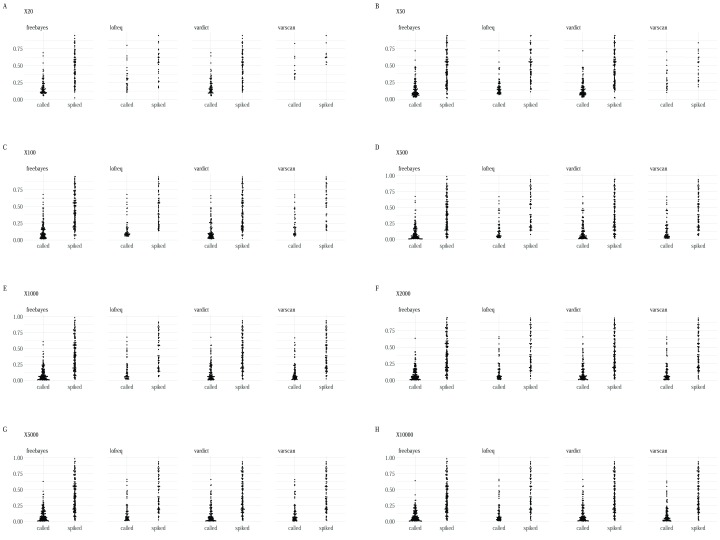
Box plots showing the distribution of frequencies between the spiked variants and the corresponding called variant for each variant caller at each coverage ( **A**– **H**) for the first dataset.

FreeBayes identified the majority of variants ( Figure 3) across all the datasets although it was characterised by a substantial trade-off between sensitivity and precision in artificial dataset 1 ( Figure 4) in addition to a high false positive rate relative to the other minority variant callers observed in datasets 1 and 3. Regardless, Freebayes reported comparatively better sensitivity relative to the rest of the tools. Lofreq was the most conservative of the evaluated callers and it missed majority of variants across all the three datasets. In addition, Lofreq’s sensitivity in coverages above 100 did not differ in a substantial way ( Figure 4). Vardict performance increased with read coverage but not by a great magnitude compared to FreeBayes and Varscan. Its performance across different datasets was more consistent relative to other tools. Overall, we observed lower reported frequency in called minority variants compared with spiked frequencies ( Figure 5). This observation was consistent in all the datasets from all the variant callers.

## Discussion

Detecting and reporting minority variant calls is challenging, given that low frequency calls occur at a frequency that is the same as error generated from sequencing and PCR reactions. Recent studies have linked the sharing of minority variants with transmission patterns, and hence it is important to distinguish actual minor variants from spurious variant calls. Several minority variants callers use different detection algorithms and statistics, each of which attempt to optimize an aspect of the variant calling process. Therefore, there could be disparities between what is reported by a given minority variant caller, given datasets of varying sequencing depths and error profiles.

This study aimed to identify the proportion of positions that were recognized as variants by a set of tools using three artificial datasets of varying coverage and error profiles. Concordance accuracy and inter-caller concordance measures were dependent on the sample coverage and error profile.

Sensitivity for the majority of the tools was positively correlated with depth of coverage and similarly observed previously ( Spencer *et al.*, 2014) in a study that investigated performance in methods used to detect low-frequency variants. It is important to note that the tools provide different performance metrics depending on the variant’s threshold ( Supplementary File 5). In the first artificial dataset, VarDict detected true positive variants with comparably good performance (sensitivity 43.4% – 72.3%), though it was marginally invariant to changes in average coverage above 20. VarDict has in-built features that could contribute to its efficient performance. It is able to activate an “amplicon calling mode” that filters out amplicon biased variants and mispaired primers as PCR artefacts. A similar pattern was observed with LoFreq, where sensitivity was not significantly affected by depth of coverage. VarScan2 was more affected by coverage and maintained average sensitivity (6.6% – 58.4%). Applying filters in VarScan has been reported to improve sensitivity by reducing number of false positives ( Hofmann *et al.*, 2017; Koboldt *et al.*, 2013). FreeBayes’ trade-off between sensitivity and precision was also reported by other studies ( Hwang *et al.*, 2015; Sandmann *et al.*, 2017). The use of caller-specified filters could have enhanced the sensitivity of some callers, but the option to adopt default parameters allows equivalent assessment of tool performance. Moreover, all possible combinations of tuning parameters are challenging, time-consuming and sometimes impractical.

Based on artificial reads from the second dataset, FreeBayes performed comparatively better than the other tools with a very low false positive rate and better sensitivity (46.4% – 94.6%). This suggests that FreeBayes is potentially useful in identifying minority variants when sample data comes from reads with a low error profile. This implies the error rate results are outcome of tool performance.

Specificity of a caller is its ability to correctly predict the absence of a variant. The variant callers make use of a high specificity to minimize the number of false positive calls thereby reducing post-call filtering and consequently filter out true low-frequency variants. Moreover, high accuracy measures demonstrate the reliability of the variant caller in correctly identifying true variants.

All the minority variant callers reported slightly lower frequencies in called variants compared to the frequencies in the original spiked variants ( Figure 5). This could be explained by the fact that many of the callers are tuned to report lower differences in the calls owing to stringent pre-processing criteria. A thorough investigation of this observation is therefore required. 

In absence of an explicit error model from samples of heterogeneous sequencing quality, combining at least three tools when identifying minority variants could potentially assist in filtering out errors from low frequency variants. Given that there are no definitive data and next generation sequencing pipeline standards for variant calling approaches that are specific for viruses, there are opportunities to develop robust methods and tools that strike a balance between detecting errors and true minority variants from field virus samples that present with different sequencing quality.

## Data availability

The data analysis scripts and datasets used in analysis are available from our institutional Dataverse repository: http://dx.doi.org/10.7910/DVN/ZIO43M ( Mohammed & Githinji, 2018)

Data are available under the terms of the Creative Commons Attribution 4.0 International license (CC-BY 4.0).

## Supplementary material

**Supplementary File 1: A description of data generation methods and command line tools used to create the artificial datasets.**

**Supplementary File 2: FastQC metrics for sample used to generate simulated reads for the second dataset.**

**Supplementary File 3: FastQC quality profile for sample used to generate simulated reads for the third dataset.**

**Supplementary File 4: Tool-specific parameter settings for variant calling**

**Supplementary File 5:** Figures showing the performance of variant calling for minority variants that were inserted at different thresholds, standard (>25%), moderate (5-25%) and low (<5%).

**Supplementary Figure 1: Proportion of fully concordant positions with respect to sample coverage in dataset1.** (A) Represents the proportion (y-axis) of fully concordant variants with respect to read coverage (x-axis) and (B) shows the proportion of variants positions that could not be identified by any minority caller at each level of sample coverage.

**Supplementary Figure 2: UpSetR plots showing the concordance between called variants and the respective variant callers using the first artificial dataset.** The intersection size illustrates the number of variants in each intersection set. The horizontal axis shows the combination matrix identifying the intersections. A single filled circle represents a unique set of variants. Connected lines depict shared variants (intersections) among the variant callers. Intersection size was positively correlated with average coverage at (a) 20X, (b) 50X, (c) 100X, (d) 500X, (e) 1000X, (f) 2000X, (g) 5000X and (h) 10,000X.

**Supplementary Tables 1-4.**

**Supplementary Table 1:** 166 artificially generated nucleotide mutations with frequencies from 0-1.

**Supplementary Table 2:** 156 artificially generated nucleotide mutations with frequencies below 0.5.

**Supplementary Table 3: A breakdown of performance metrics of variant callers evaluated using the second dataset incorporated with an error profile derived from the set of reads used to assemble the reference genome.** The samples represent simulated dataset of varying average depth of coverage. True positive (TP), true negative (TN), false positive (FP) and false negatives (FN) were used to calculate performance metrics of each caller. FPR – False positive rate.

**Supplementary Table 4: A breakdown of performance metrics of variant callers evaluated using the third dataset generated with an error profile from a poorly sequenced sample.** The samples represent simulated dataset of varying average depth of coverage. True positive (TP), true negative (TN), false positive (FP) and false negatives (FN) were used to calculate performance metrics of each caller. FPR – False positive rate.

## References

[ref-1] Domingo E Sheldon J Perales C: Viral quasispecies evolution. *Microbiol Mol Biol Rev.* 2012;76(2):159–216. 10.1128/mmbr.05023-11 22688811 PMC3372249

[ref-2] Eigen M McCaskill J Schuster P: Molecular Quasi-Species. *J Phys Chem.* 1988;92(24):6881–6891. 10.1021/j100335a010

[ref-3] Ewing AD Houlahan KE Hu Y: Combining tumor genome simulation with crowdsourcing to benchmark somatic single-nucleotide-variant detection. *Nat Methods.* 2015;12(7):623–630. 10.1038/nmeth.3407 25984700 PMC4856034

[ref-4] Henn MR Boutwell CL Charlebois P: Whole genome deep sequencing of HIV-1 reveals the impact of early minor variants upon immune recognition during acute infection. *PLoS Pathog.* 2012;8(3):e1002529. 10.1371/journal.ppat.1002529 22412369 PMC3297584

[ref-5] Hofmann AL Behr J Singer J: Detailed simulation of cancer exome sequencing data reveals differences and common limitations of variant callers. *BMC Bioinformatics.* 2017;18(1):8. 10.1186/s12859-016-1417-7 28049408 PMC5209852

[ref-6] Holland JJ De La Torre JC Steinhauer DA: RNA virus populations as quasispecies. *Curr Top Microbiol Immunol.* 1992;176:1–20. 10.1007/978-3-642-77011-1_1 1600747

[ref-7] Huang HW, NISC Comparative Sequencing Program Mullikin JC: Evaluation of variant detection software for pooled next-generation sequence data. *BMC Bioinformatics.* 2015;16:235. 10.1186/s12859-015-0624-y 26220471 PMC4518579

[ref-8] Huang W Li L Myers JR: ART: a next-generation sequencing read simulator. *Bioinformatics.* 2012;28(4):593–594. 10.1093/bioinformatics/btr708 22199392 PMC3278762

[ref-9] Hwang S Kim E Lee I: Systematic comparison of variant calling pipelines using gold standard personal exome variants. *Sci Rep.* 2015;5:17875. 10.1038/srep17875 26639839 PMC4671096

[ref-10] Kinde I Wu J Papadopoulos N: Detection and quantification of rare mutations with massively parallel sequencing. *Proc Natl Acad Sci U S A.* 2011;108(23):9530–9535. 10.1073/pnas.1105422108 21586637 PMC3111315

[ref-11] Koboldt DC Chen K Wylie T: VarScan: variant detection in massively parallel sequencing of individual and pooled samples. *Bioinformatics.* 2009;25(17):2283–2285. 10.1093/bioinformatics/btp373 19542151 PMC2734323

[ref-12] Koboldt DC Larson DE Wilson RK: Using VarScan 2 for Germline Variant Calling and Somatic Mutation Detection. *Curr Protoc Bioinformatics.* 2013;44:15.4.1–17. 10.1002/0471250953.bi1504s44 25553206 PMC4278659

[ref-13] Koboldt DC Zhang Q Larson DE: VarScan 2: somatic mutation and copy number alteration discovery in cancer by exome sequencing. *Genome Res.* 2012;22(3):568–576. 10.1101/gr.129684.111 22300766 PMC3290792

[ref-14] Krøigård AB Thomassen M Laenkholm AV: Evaluation of Nine Somatic Variant Callers for Detection of Somatic Mutations in Exome and Targeted Deep Sequencing Data. *PLoS One.* 2016;11(3):e0151664. 10.1371/journal.pone.0151664 27002637 PMC4803342

[ref-15] Lai Z Markovets A Ahdesmaki M: VarDict: a novel and versatile variant caller for next-generation sequencing in cancer research. *Nucleic Acids Res.* 2016;44(11):e108. 10.1093/nar/gkw227 27060149 PMC4914105

[ref-16] Lex A Gehlenborg N Strobelt H: UpSet: Visualization of Intersecting Sets. *IEEE Trans Vis Comput Graph.* 2014;20(12):1983–1992. 10.1109/tvcg.2014.2346248 26356912 PMC4720993

[ref-17] Macalalad AR Zody MC Charlebois P: Highly sensitive and specific detection of rare variants in mixed viral populations from massively parallel sequence data. *PLoS Comput Biol.* 2012;8(3):e1002417. 10.1371/journal.pcbi.1002417 22438797 PMC3305335

[ref-18] McCrone JT Lauring AS: Measurements of Intrahost Viral Diversity Are Extremely Sensitive to Systematic Errors in Variant Calling. *J Virol.* 2016;90(15):6884–6895. 10.1128/jvi.00667-16 27194763 PMC4944299

[ref-19] Mohammed KS Githinji G: Replication Data for: Evaluating the Performance of Tools Used to Call Minority Variants from Whole Genome Short-Read Data. *Harvard Dataverse, V3.* 2018 10.7910/DVN/ZIO43M PMC6234735 30483597

[ref-20] Moya A Holmes EC González-Candelas F: The population genetics and evolutionary epidemiology of RNA viruses. *Nat Rev Microbiol.* 2004;2(4):279–288. 10.1038/nrmicro863 15031727 PMC7096949

[ref-21] Pabinger S Dander A Fischer M: A survey of tools for variant analysis of next-generation genome sequencing data. *Brief Bioinform.* 2014;15(2):256–278. 10.1093/bib/bbs086 23341494 PMC3956068

[ref-22] Poon LL Song T Rosenfeld R: Quantifying influenza virus diversity and transmission in humans. *Nat Genet.* 2016;48(2):195–200. 10.1038/ng.3479 26727660 PMC4731279

[ref-23] Sandmann S de Graaf AO Karimi M: Evaluating Variant Calling Tools for Non-Matched Next-Generation Sequencing Data. *Sci Rep.* 2017;7:43169. 10.1038/srep43169 28233799 PMC5324109

[ref-24] Spencer DH Tyagi M Vallania F: Performance of common analysis methods for detecting low-frequency single nucleotide variants in targeted next-generation sequence data. *J Mol Diagn.* 2014;16(1):75–88. 10.1016/j.jmoldx.2013.09.003 24211364 PMC3873500

[ref-25] Stack JC Murcia PR Grenfell BT: Inferring the inter-host transmission of influenza A virus using patterns of intra-host genetic variation. *Proc Biol Sci.* 2013;280(1750):20122173. 10.1098/rspb.2012.2173 23135678 PMC3574438

[ref-26] Vignuzzi M Stone JK Arnold JJ: Quasispecies diversity determines pathogenesis through cooperative interactions in a viral population. *Nature.* 2006;439(7074):344–348. 10.1038/nature04388 16327776 PMC1569948

[ref-27] Watson SJ Welkers MR Depledge DP: Viral population analysis and minority-variant detection using short read next-generation sequencing. *Philos Trans R Soc Lond B Biol Sci.* 2013;368(1614):20120205. 10.1098/rstb.2012.0205 23382427 PMC3678329

[ref-28] Wilm A Aw PP Bertrand D: LoFreq: a sequence-quality aware, ultra-sensitive variant caller for uncovering cell-population heterogeneity from high-throughput sequencing datasets. *Nucleic Acids Res.* 2012;40(22):11189–11201. 10.1093/nar/gks918 23066108 PMC3526318

[ref-29] Worby CJ Lipsitch M Hanage WP: Shared Genomic Variants: Identification of Transmission Routes Using Pathogen Deep-Sequence Data. *Am J Epidemiol.* 2017;186(10):1209–1216. 10.1093/aje/kwx182 29149252 PMC5860558

[ref-30] Yang X Charlebois P Macalalad A: V-Phaser 2: variant inference for viral populations. *BMC Genomics.* 2013;14:674. 10.1186/1471-2164-14-674 24088188 PMC3907024

[ref-31] Zhang F Flaherty P: Variational inference for rare variant detection in deep, heterogeneous next-generation sequencing data. *BMC Bioinformatics.* 2017;18(1):45. 10.1186/s12859-016-1451-5 28103803 PMC5244592

[ref-32] Zukurov JP do Nascimento-Brito S Volpini AC: Estimation of genetic diversity in viral populations from next generation sequencing data with extremely deep coverage. *Algorithms Mol Biol.* 2016;11:2. 10.1186/s13015-016-0064-x 26973707 PMC4788855

