# Supplementary material for: Evaluating the performance of tools used to call minority variants from whole genome short-read data
Source: Wellcome Open Res. 2018 Sep 13;3:21. Originally published 2018 Mar 5. [Version 2] doi: 10.12688/wellcomeopenres.13538.2 (PMC6234735; doi:10.12688/wellcomeopenres.13538.2)
Supplement: Supplementary file 1 [file wellcomeopenres-3-16071-s0000.tgz › d5d3f712-81c0-495c-88eb-c65da9dabd94.docx]

**Supplementary File 1**

1. **Artificial data**
   1. **Generation of artificial datasets**

Simulation of artificial samples for each dataset was performed using ART Illumina (2.5.8). installed on a CentOS 7 Linux computing cluster. The following commands were executed to generate each sample for each dataset. This was run at each level of coverage by setting the –f parameter (20, 50, 100, 500, 1000, 2000, 5000, 10000) resulting in 8 samples per dataset.

art_illumina -sam i- $ref_seq -p -l 150 -ss MSv1 -f 20 -m 800 -s 10 –o output1.sam

Prior to simulation, read quality profiles were determined from uncompressed fastqc raw reads of two sets of sequences based on the FastQC metrics, then two error profiles were generated from the two Illumina paired-end sequenced samples and the previous command was modified to incorporate those error models.

art_illumina -sam i- $ref_seq -p -l 150 -ss MSv1 -f 20 -m 800 -s 10 -1 miseq250.R1.txt -2 miseq.R2.txt -o output2.sam

The resulting SAM files were converted to BAM files, sorted and indexed using SAMtools 1.3.1.

- 1. **Generation of variants**

We generated a list of uniform random positions, nucleotides at various frequencies (Supplementary Table 1). We selected the variants such that the nucleotide did not match with the reference nucleotide at the preferred position. The variants were added (“spiked”) to the artificially generated datasets (in BAM format) using BAMSurgeon (Ewing *et al*., 2015).

With the list of variants generated, BAMSurgeon introduced the SNVs to the simulated Illumina MiSeq data using the following command for each sample coverage;

module load python 2.7.8, picard 2.3.0, samtools 1.3.1 , velvet 1.2.10-kmer64, exonerate 2.4.0, bwa 0.7.13-r1126

addsnv.py –v variant.list –f sim.sample1.sorted.bam –r 9465113.fa –o sample1.snv.bam - - maxdepth 25000
