# Supplementary material for: Evaluating the performance of tools used to call minority variants from whole genome short-read data
Source: Wellcome Open Res. 2018 Sep 13;3:21. Originally published 2018 Mar 5. [Version 2] doi: 10.12688/wellcomeopenres.13538.2 (PMC6234735; doi:10.12688/wellcomeopenres.13538.2)
Supplement: Supplementary file 3 [file wellcomeopenres-3-16071-s0002.tgz › 75206cf9-5c59-42ed-bbb2-06d918076259.pdf]

FastQC summary report

Basic information

```
basic.info1
```

```
##                file_name              encoding               file_type
## 1 H_4002_18_02_1.fastq.gz Sanger / Illumina 1.9 Conventional base calls
##   sequence_length poor_quality_seqs total_sequences gc_percent
## 1             150                 0          450944         46
```

```
basic.info2
```

```
##                file_name              encoding               file_type
## 1 H_4002_18_02_2.fastq.gz Sanger / Illumina 1.9 Conventional base calls
##   sequence_length poor_quality_seqs total_sequences gc_percent
## 1             150                 0          450944         47
```

Per base sequence quality

Sequence length distribution

Some high throughput sequencers generate sequence fragments of uniform length, but others can contain reads of wildly varying lengths. Even within uniform length libraries some pipelines will trim sequences to remove poor quality base calls from the end.

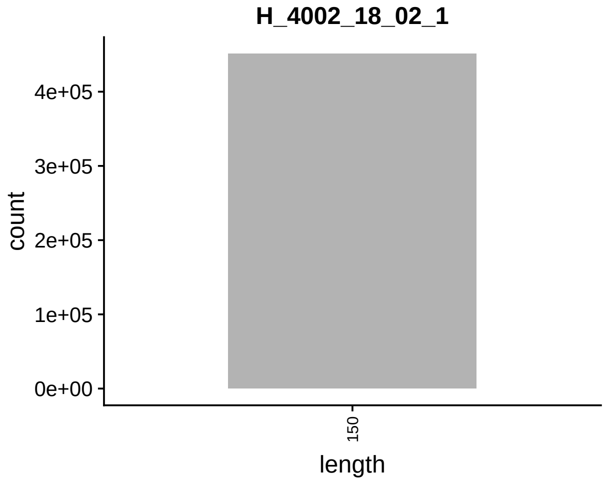

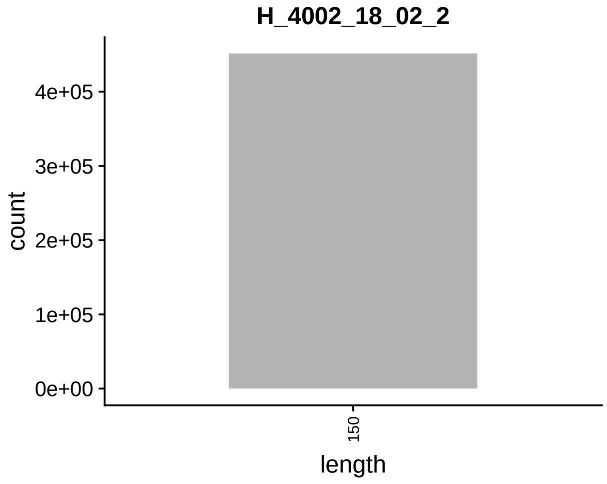

Per base quality scores

The per sequence quality score report allows you to see if a subset of your sequences have universally low quality values. It is often the case that a subset of sequences will have universally poor quality, often because

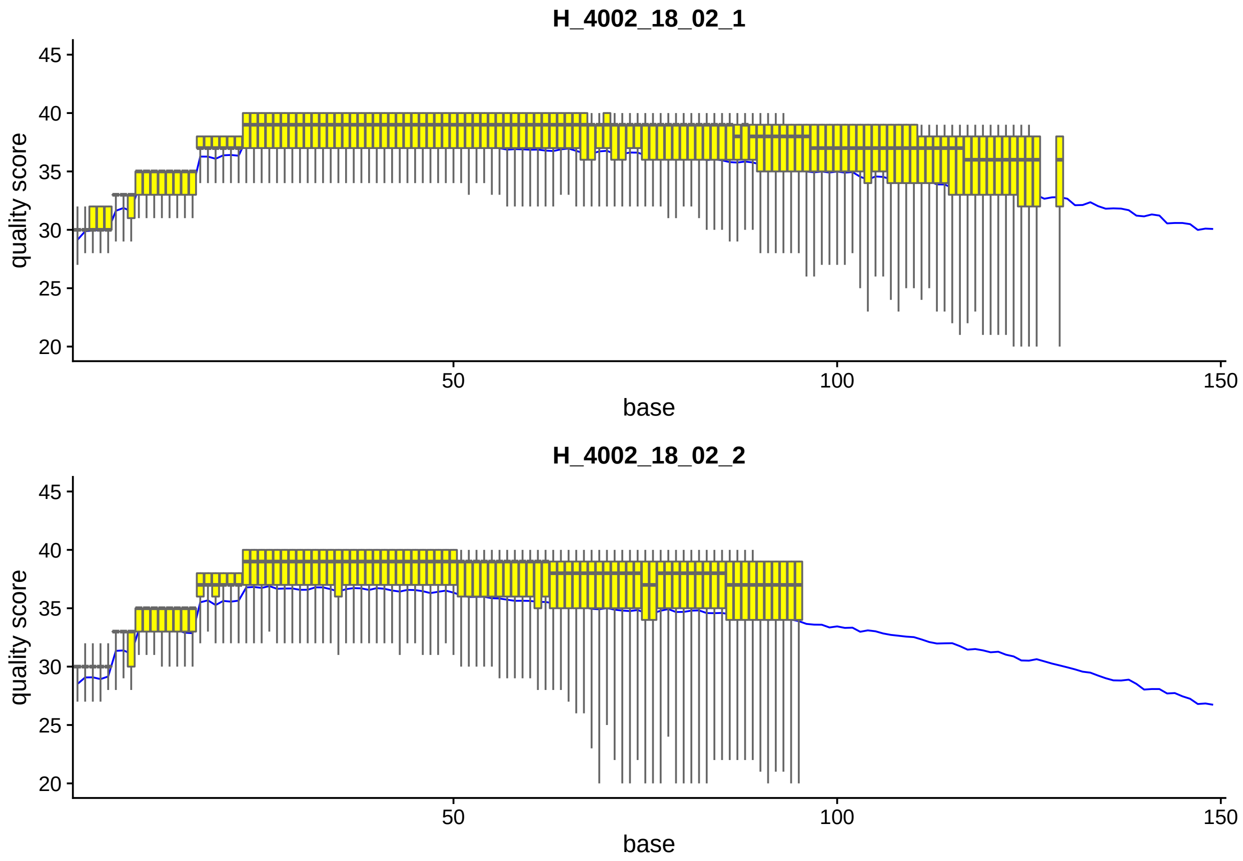

Figure 1: Per base sequence quality

they are poorly imaged (on the edge of the field of view etc), however these should represent only a small percentage of the total sequences.

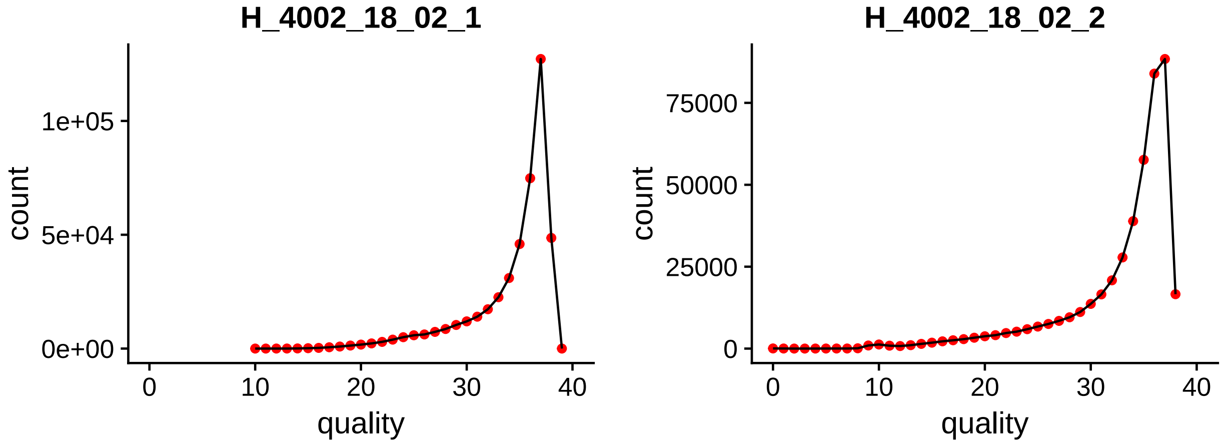

If a significant proportion of the sequences in a run have overall low quality then this could indicate some kind of systematic problem - possibly with just part of the run (for example one end of a flowcell).

Per base sequence content

Per Base Sequence Content plots out the proportion of each base position in a file for which each of the four normal DNA bases has been called. n a random library you would expect that there would be little to no difference between the different bases of a sequence run, so the lines in this plot should run parallel with each other. The relative amount of each base should reflect the overall amount of these bases in your genome, but in any case they should not be hugely imbalanced from each other.

Number of ambiguous bases per position

A plot of the percentage of base calls at each position for which an N was called. It's not unusual to see a very low proportion of Ns appearing in a sequence, especially nearer the end of a sequence. However, if this proportion rises above a few percent it suggests that the analysis pipeline was unable to interpret the data well enough to make valid base calls.

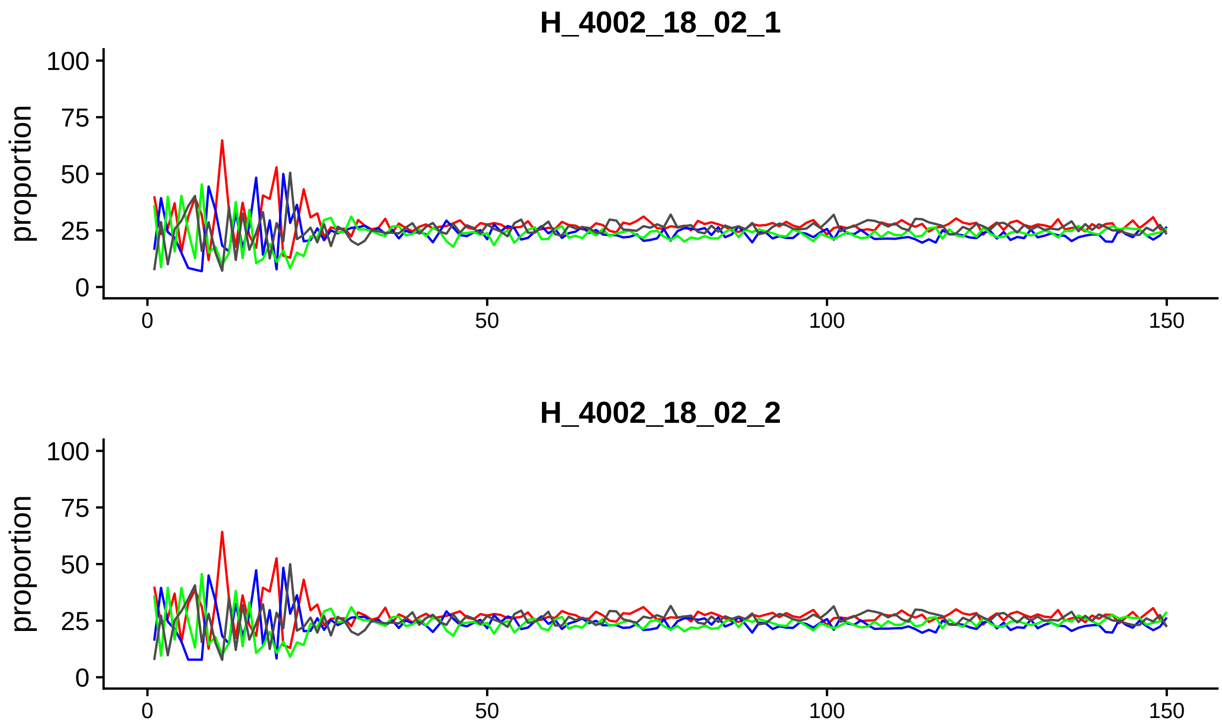

Figure 2: Per base sequence content

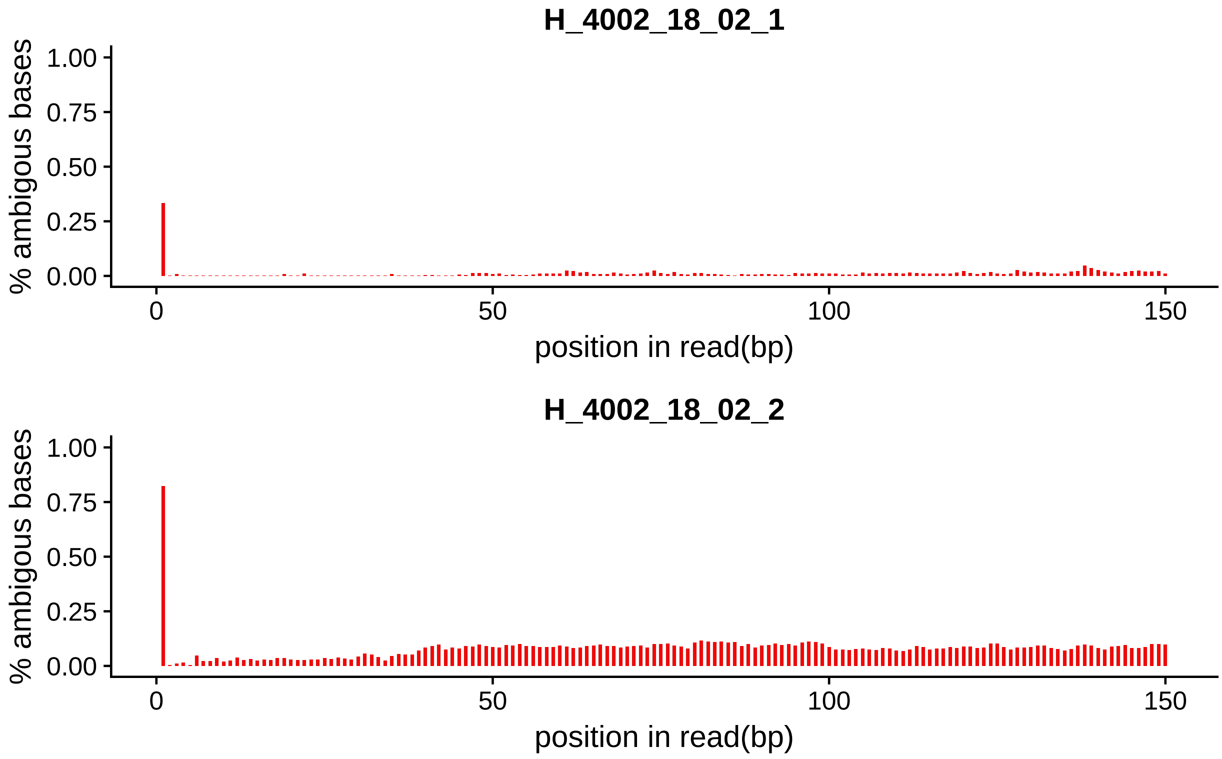

Distribution of GC content in each sequence

In a normal random library you would expect to see a roughly normal distribution of GC content where the central peak corresponds to the overall GC content of the underlying genome. Since we don't know the the GC content of the genome the modal GC content is calculated from the observed data and used to build a reference distribution. An unusually shaped distribution could indicate a contaminated library or some other kinds of biased subset. A normal distribution which is shifted indicates some systematic bias which is independent of base position. If there is a systematic bias which creates a shifted normal distribution then this won't be flagged as an error by the module since it doesn't know what your genome's GC content should be.

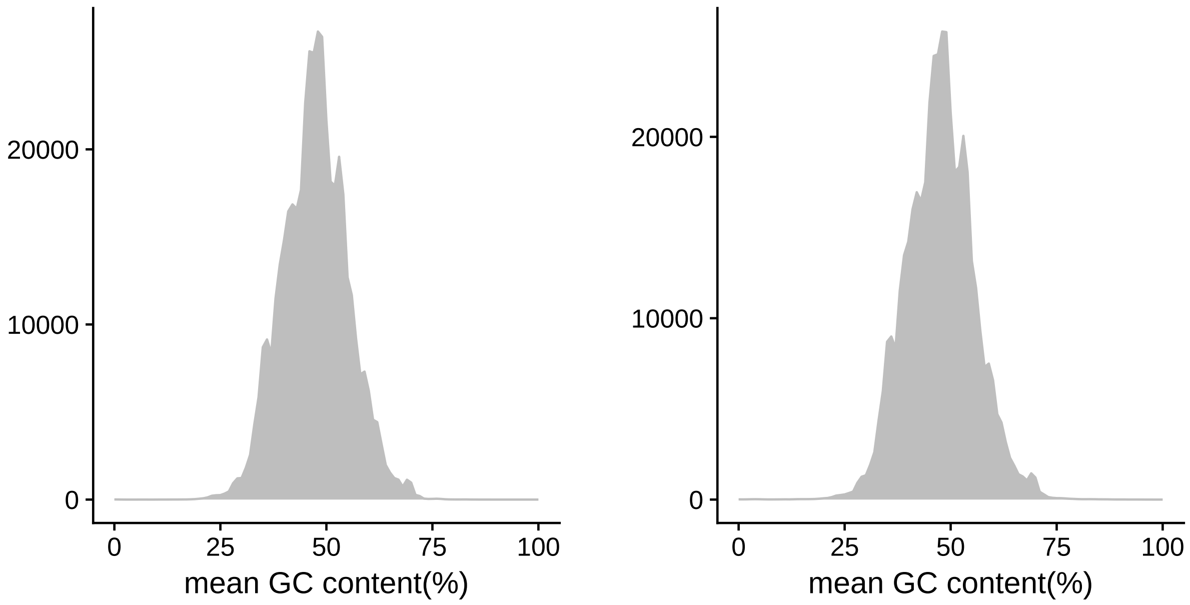

Sequence duplication

The blue line represents the counts of all the sequences that are duplicated at a given rate. The percentage is computed relative to the total number of reads.

The red line represents the number of distinct sequences that are duplicated at a given rate. The percentage is computed relative to the total number of distinct sequences in the data.

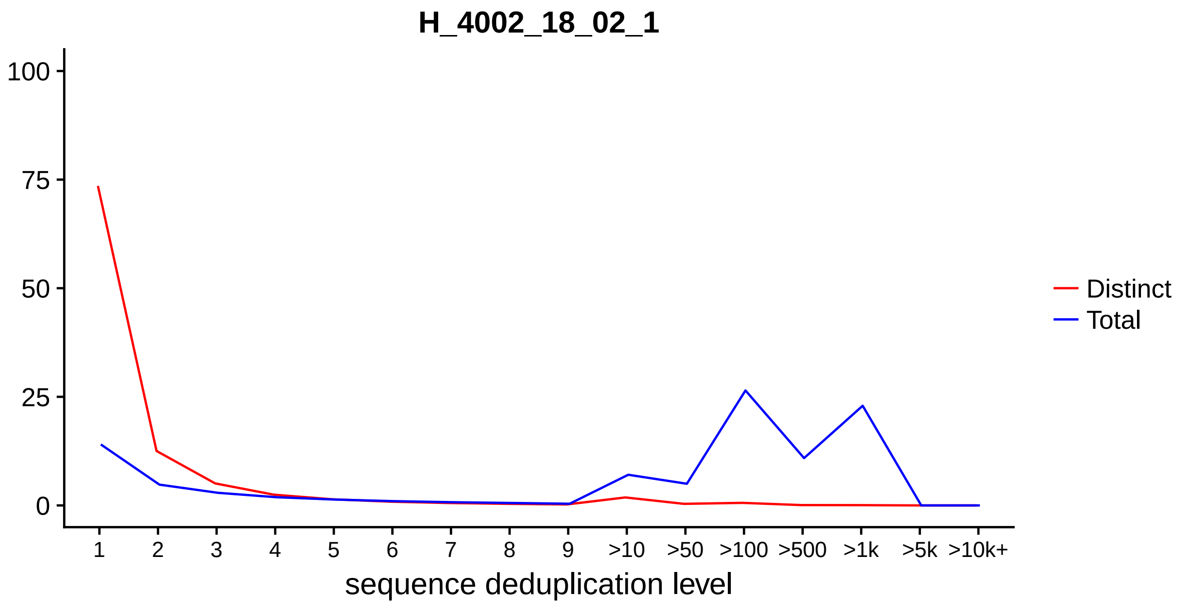

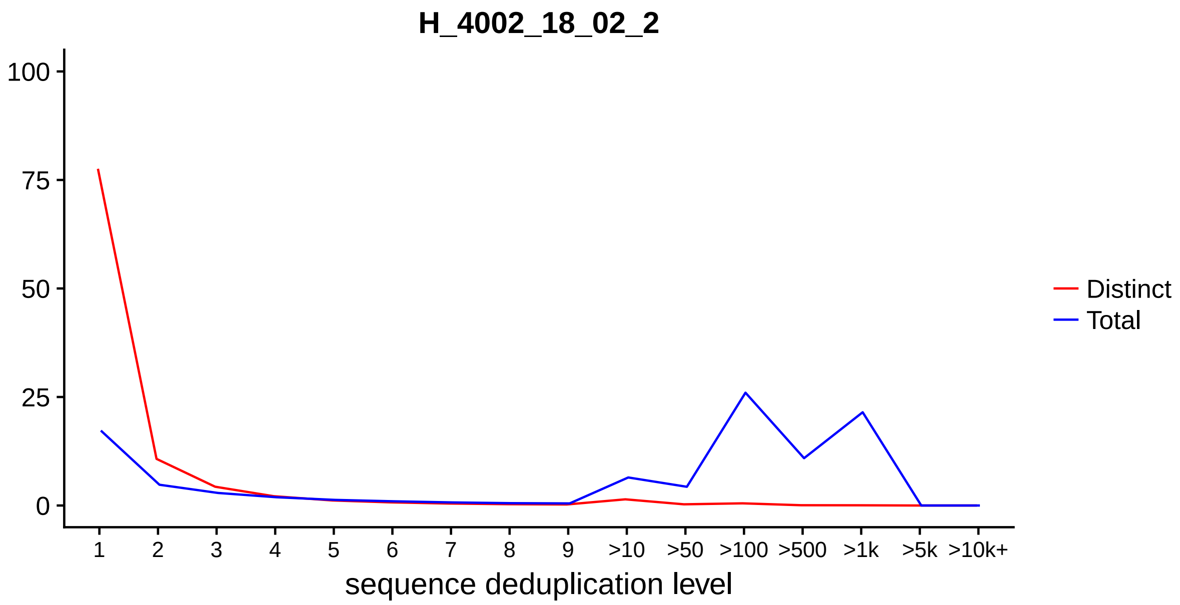

In a properly diverse library most sequences should fall into the far left of the plot in both the red and blue lines. A general level of enrichment, indicating broad oversequencing in the library will tend to flatten the lines, lowering the low end and generally raising other categories. More specific enrichments of subsets, or the presence of low complexity contaminants will tend to produce spikes towards the right of the plot. These high duplication peaks will most often appear in the red trace as they make up a high proportion of the original library, but usually disappear in the blue trace as they make up an insignificant proportion of the deduplicated set. If peaks persist in the blue trace then this suggests that there are a large number of different highly duplicated sequences which might indicate either a contaminant set or a very severe technical duplication. The module also calculates an expected overall loss of sequence were the library to be deduplicated. This headline figure is shown at the top of the plot and gives a reasonable impression of the potential overall level of loss.
