# Supplementary material for: Evaluating the performance of tools used to call minority variants from whole genome short-read data
Source: Wellcome Open Res. 2018 Sep 13;3:21. Originally published 2018 Mar 5. [Version 2] doi: 10.12688/wellcomeopenres.13538.2 (PMC6234735; doi:10.12688/wellcomeopenres.13538.2)
Supplement: Supplementary file 4 [file wellcomeopenres-3-16071-s0003.tgz › 3a8f989c-12e7-4726-9f3f-a1aeaeb90280.docx]

**Supplementary File 4**

**Variant calling parameters**

***FreeBayes***

We used Freebayes to call variants from aligned short-read data. It required a BAM file input, a reference FASTA file and it provided a VCF output. The minimum alternate fraction was set at 0.005.

Freebayes -i -X -U –min-coverage 10 -F 0.005 -f reference.fa sample.bam sample.freebayes.vcf

***LoFreq***

We used LoFreq to call variants while making use of multiple processors. The parallel wrapper thread number was set to run 10 threads per sequence/chromosome listed in the header. The reference file was indexed, the input was a BAM file and the output, a VCF file.

lofreq faidx reference.fa

lofreq call-parallel -pp-threads 10 -f reference.fa -O sample.lofreq.vcf sample.bam

***VarDict***

VarDict accepted an indexed BAM file for input and an indexed reference FASTA file while the output was a text file. The indicators for the chromosome names being numbers and printing row headers describing columns were selected. The threshold for allele frequency was set at 0.005.

Verdict -C -G reference.fa -f 0.005 -N samplename -b sample.bam -h -v -R reference sample.vardict.vcf

***Varscan2***

We used Varscan2 to call variants from a SAMtools mpileup format and the output, a VCF file. The minimum variant allele frequency threshold was adjusted to 0.005.

*samtools mpileup -f reference sample.bcf*

*java -jar varscan.jar mpileup2snp sample.bcf –min-var-freq 0.005 –output-vcf sample.varscan.vcf*
