# Supplementary figures and images for: Evaluating the performance of tools used to call minority variants from whole genome short-read data
Source: Wellcome Open Res. 2018 Sep 13;3:21. Originally published 2018 Mar 5. [Version 2] doi: 10.12688/wellcomeopenres.13538.2 (PMC6234735; doi:10.12688/wellcomeopenres.13538.2)

A

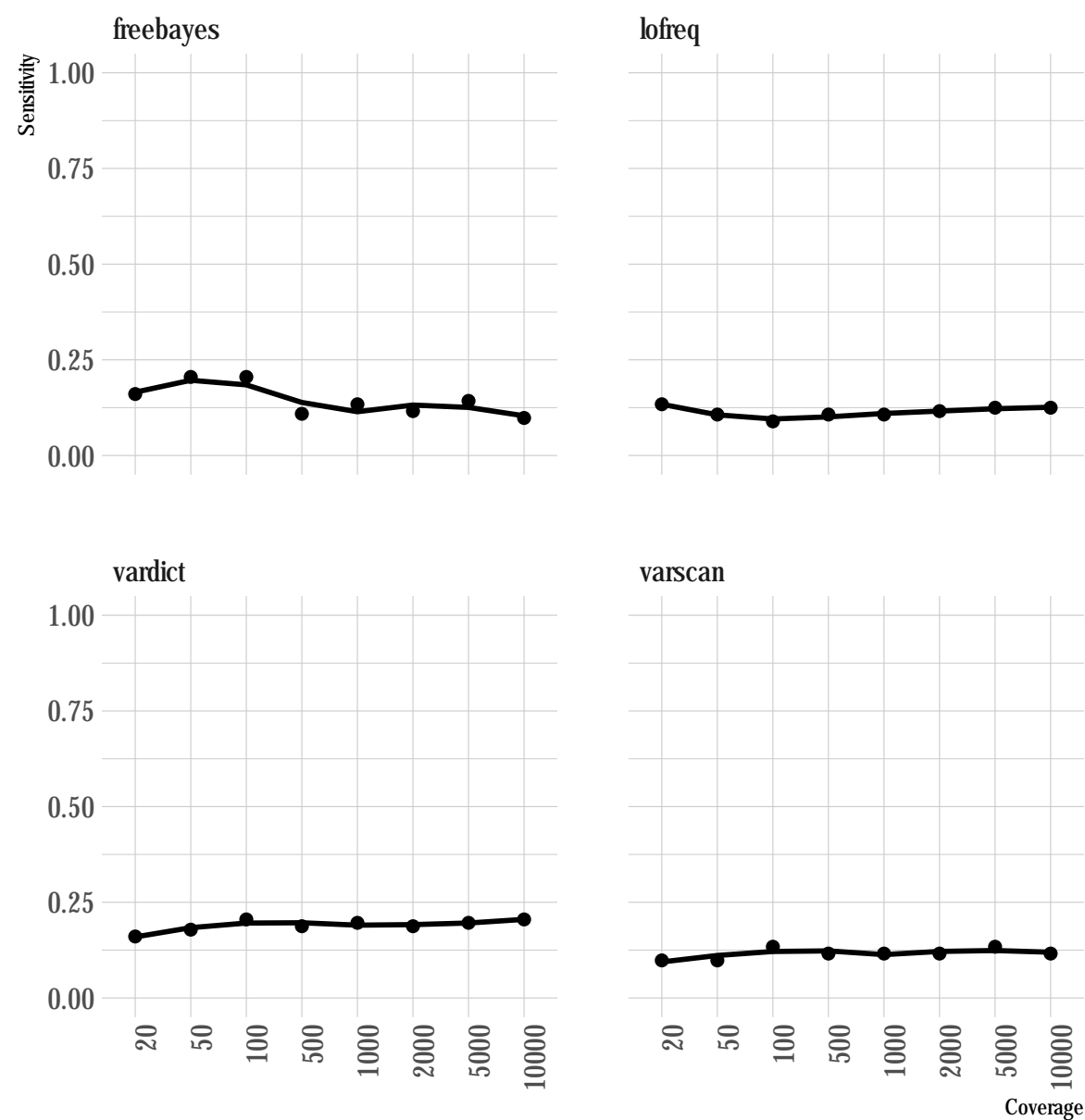

B

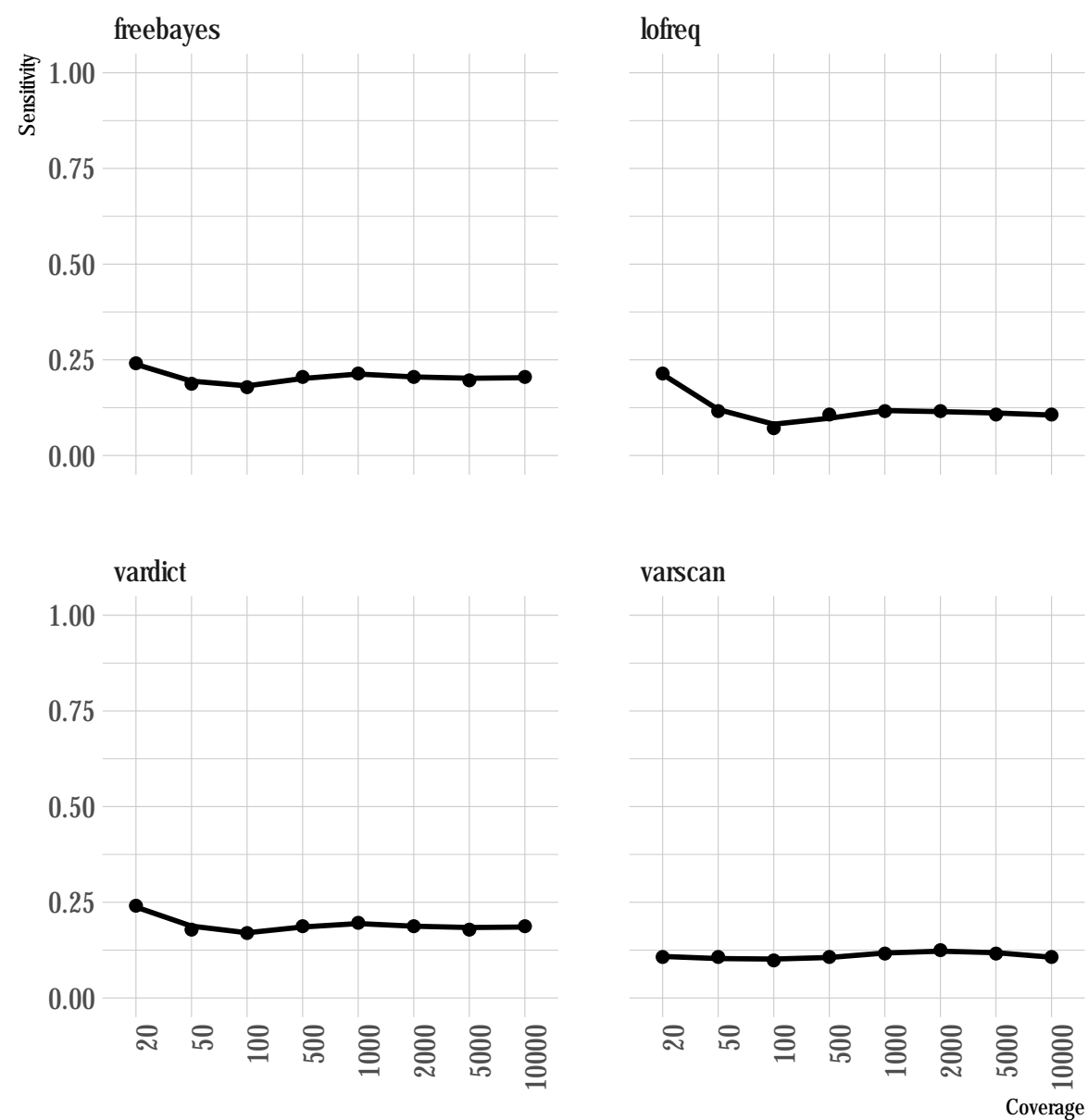

C

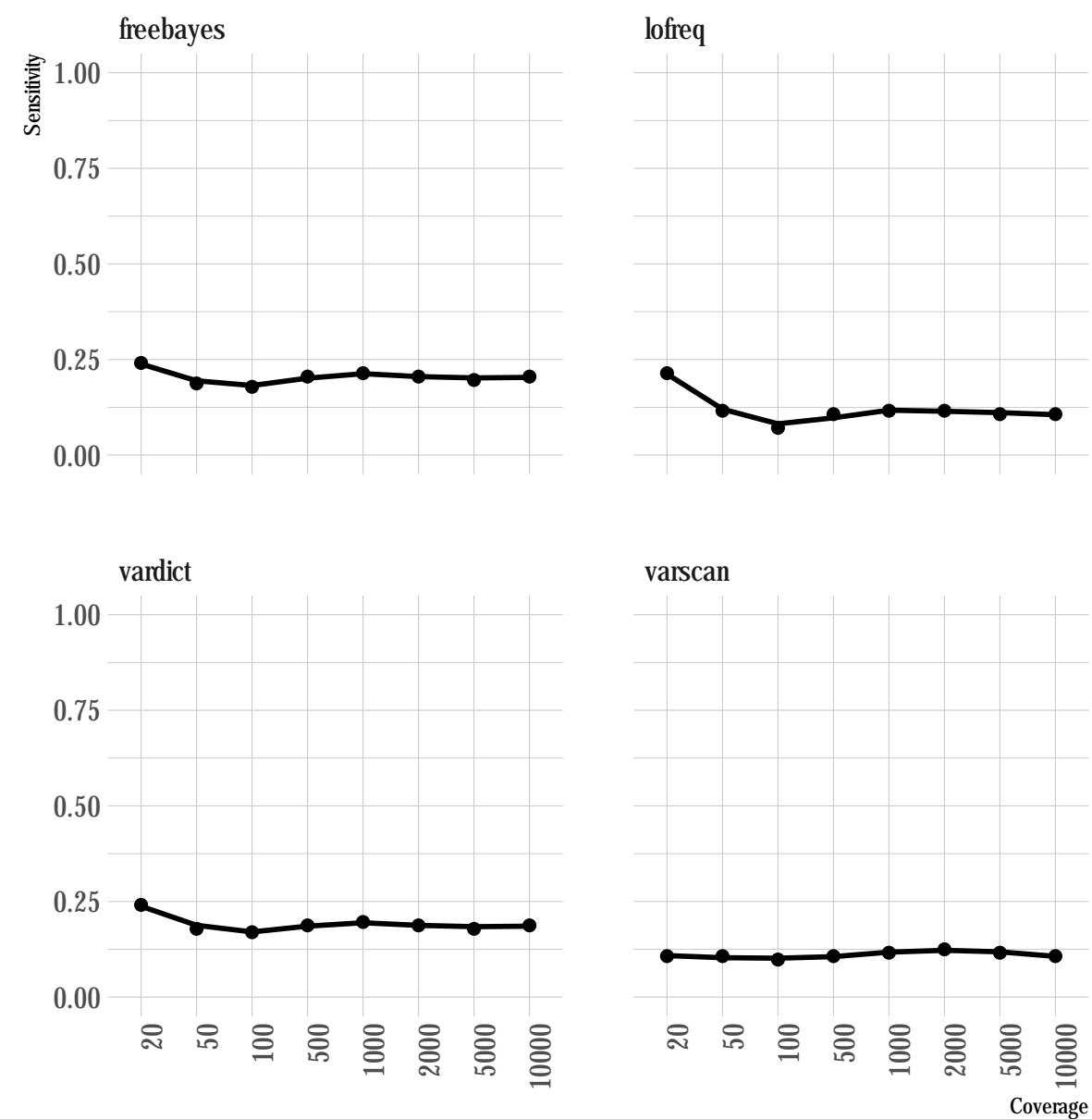

D

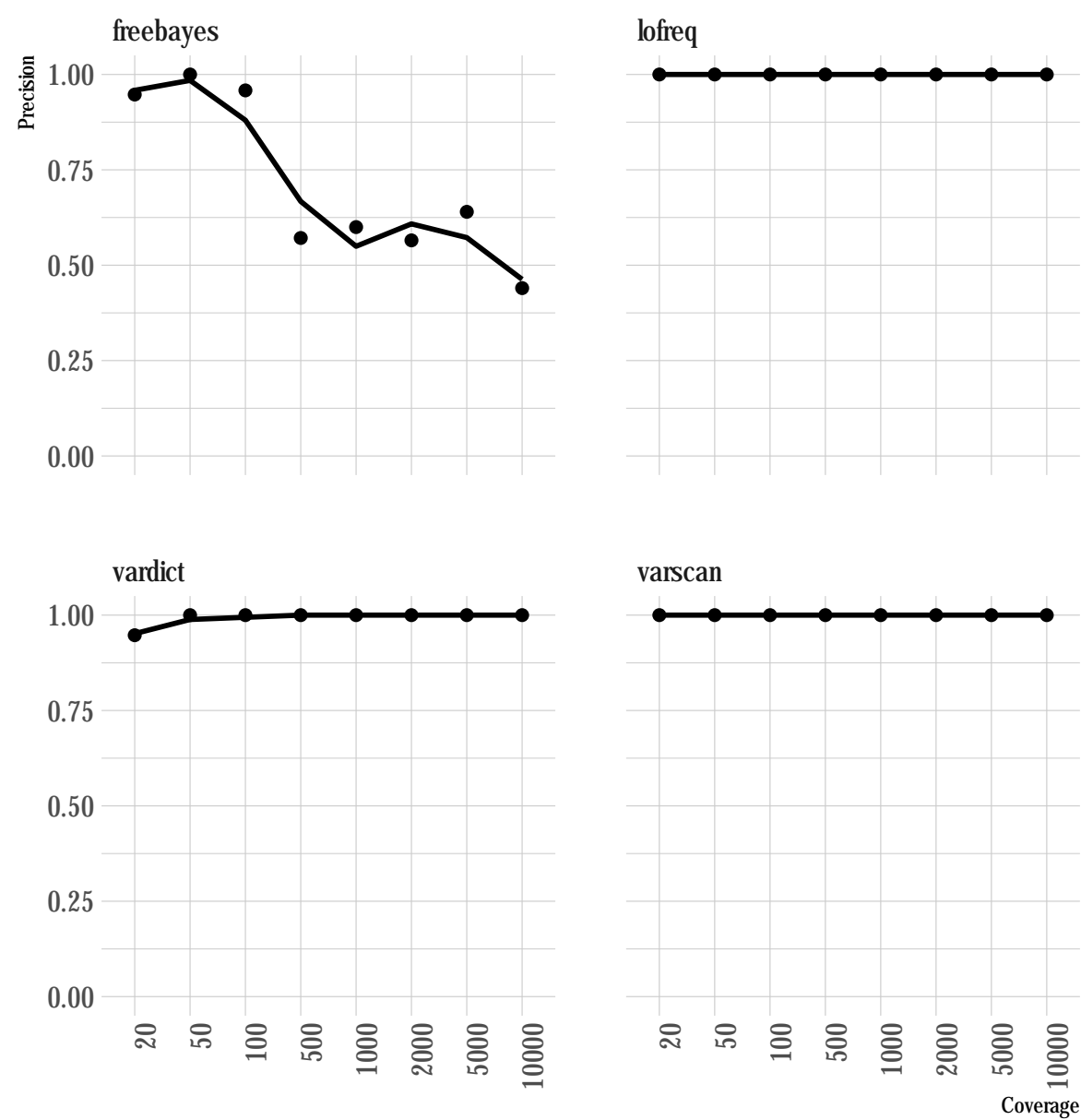

E

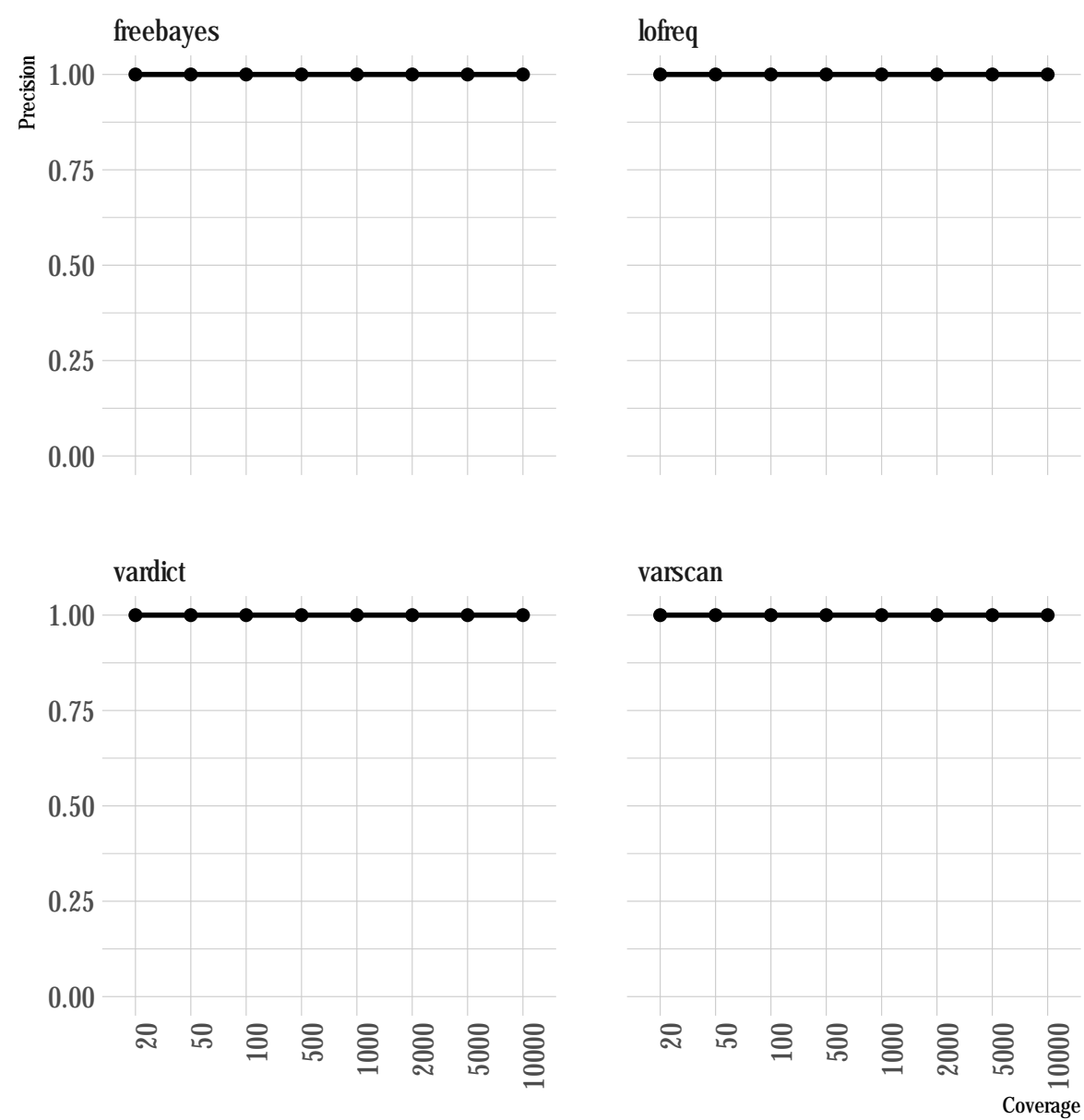

F

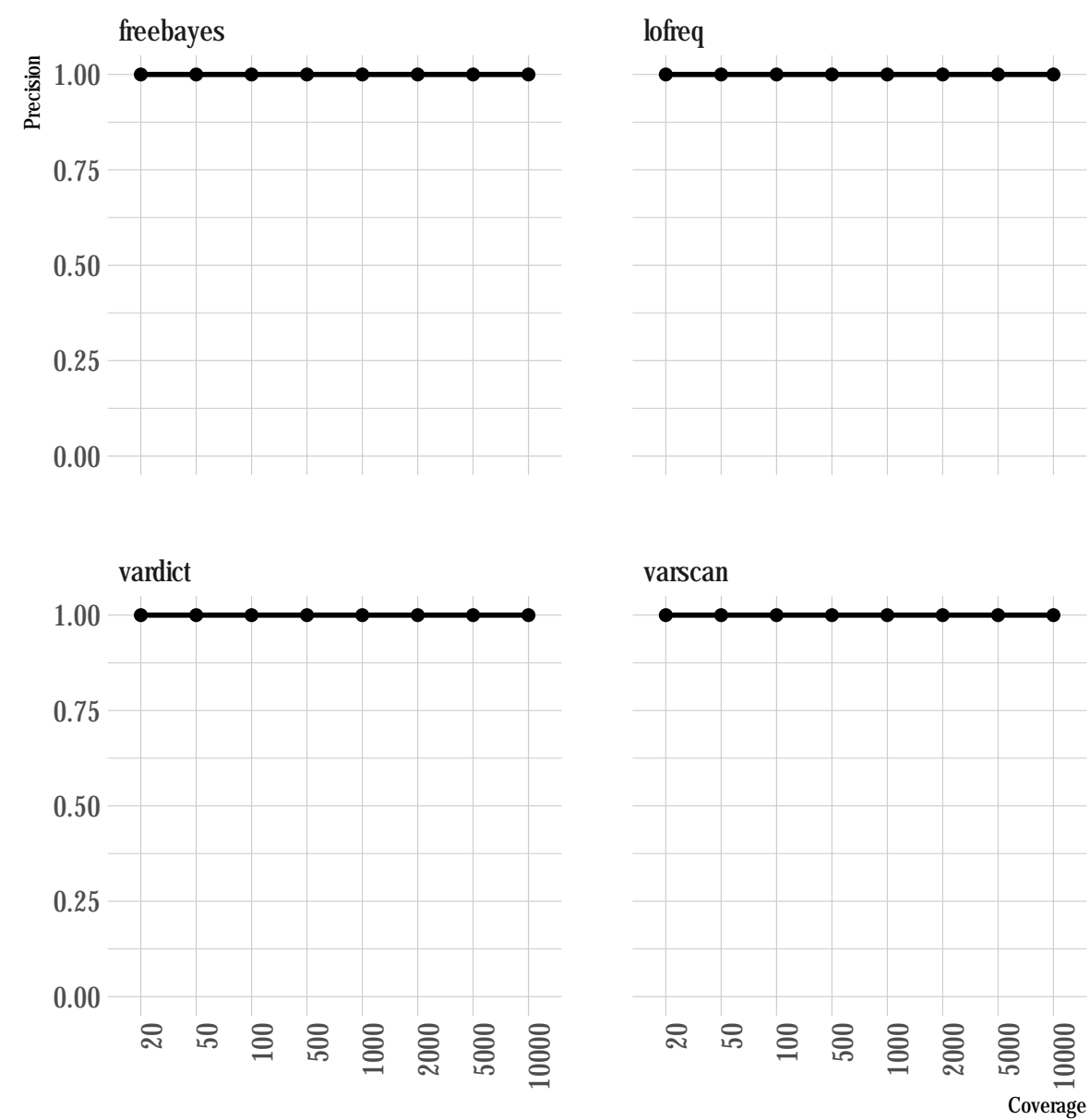

A

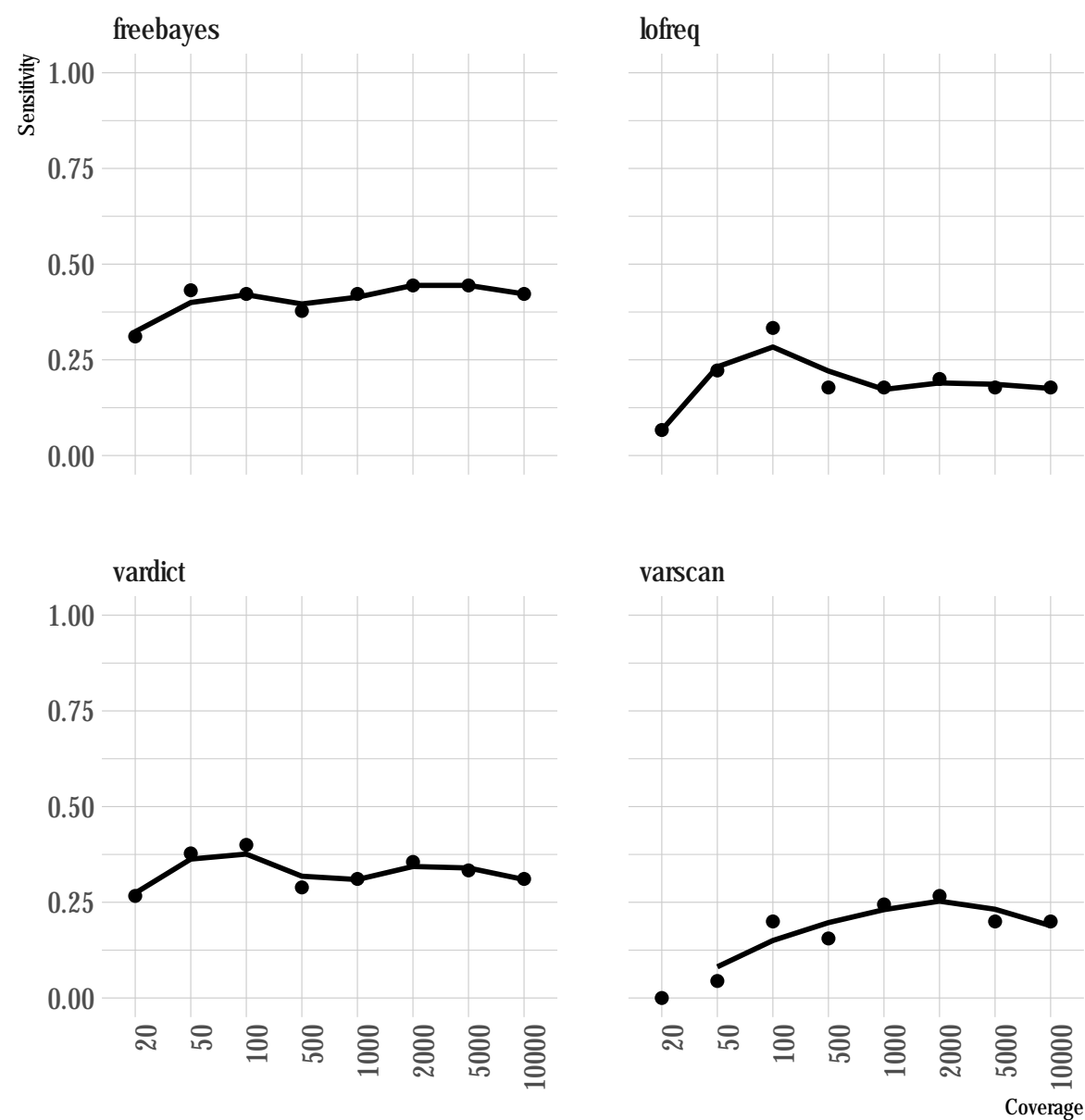

B

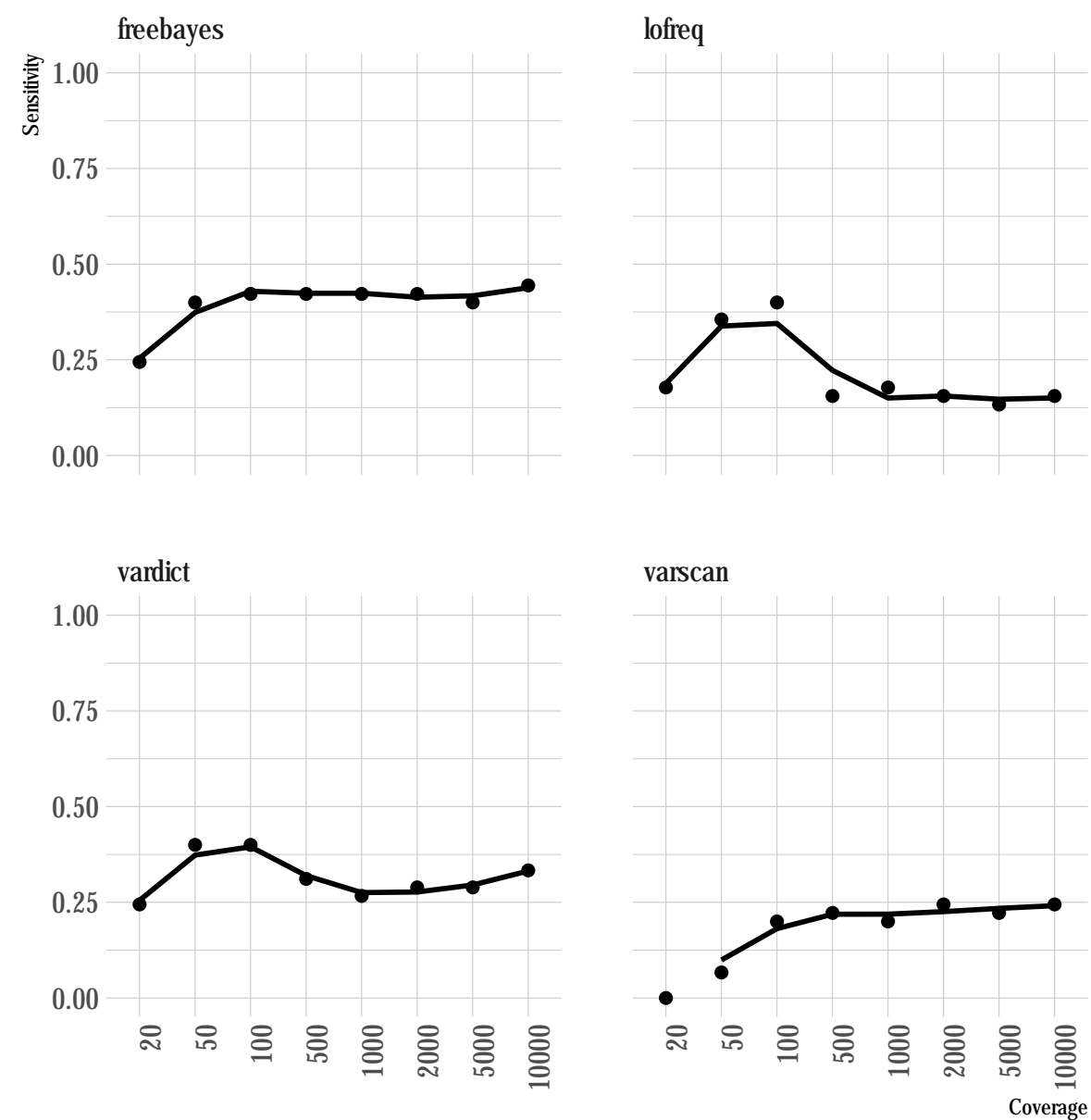

C

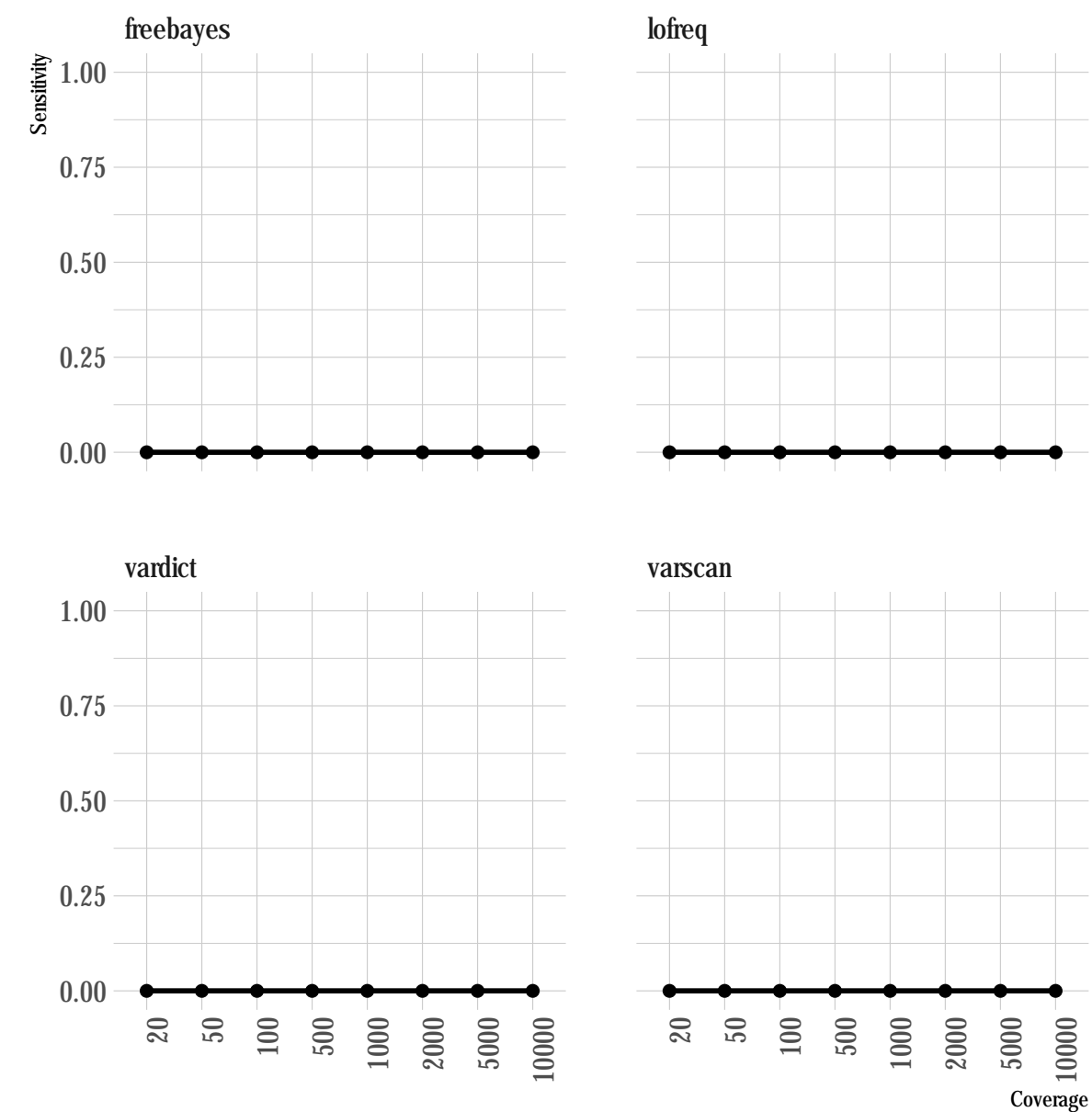

D

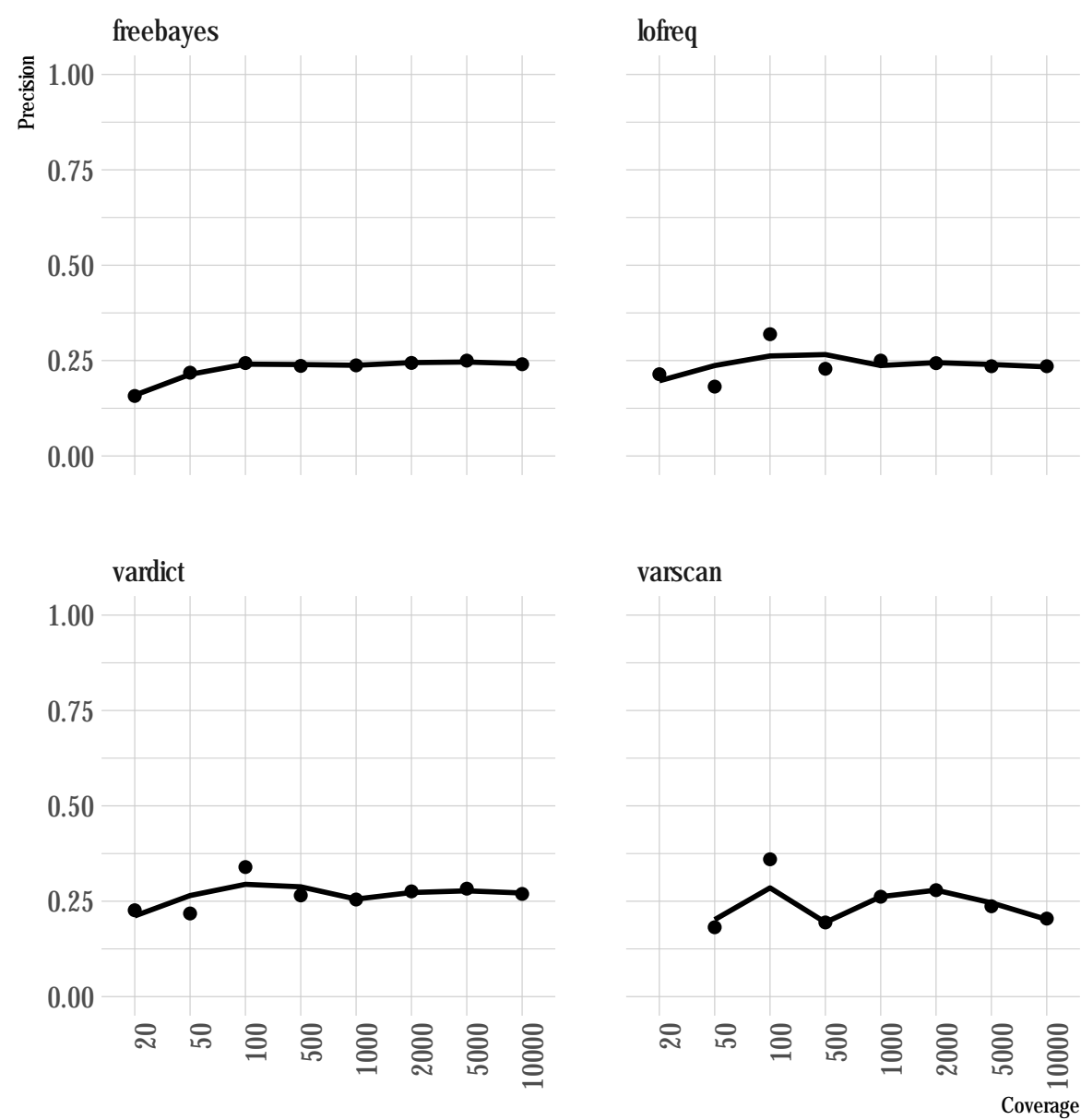

E

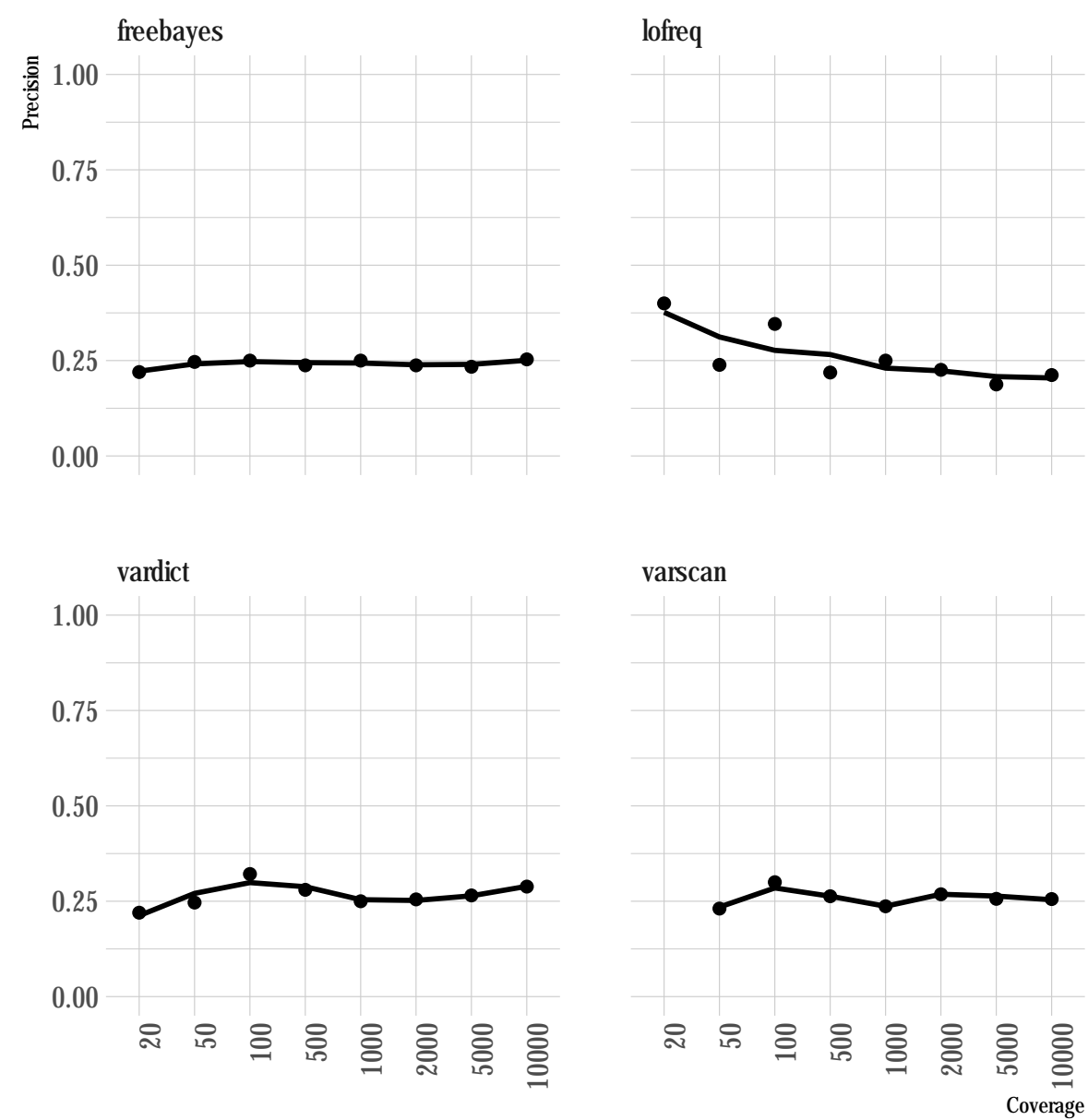

F

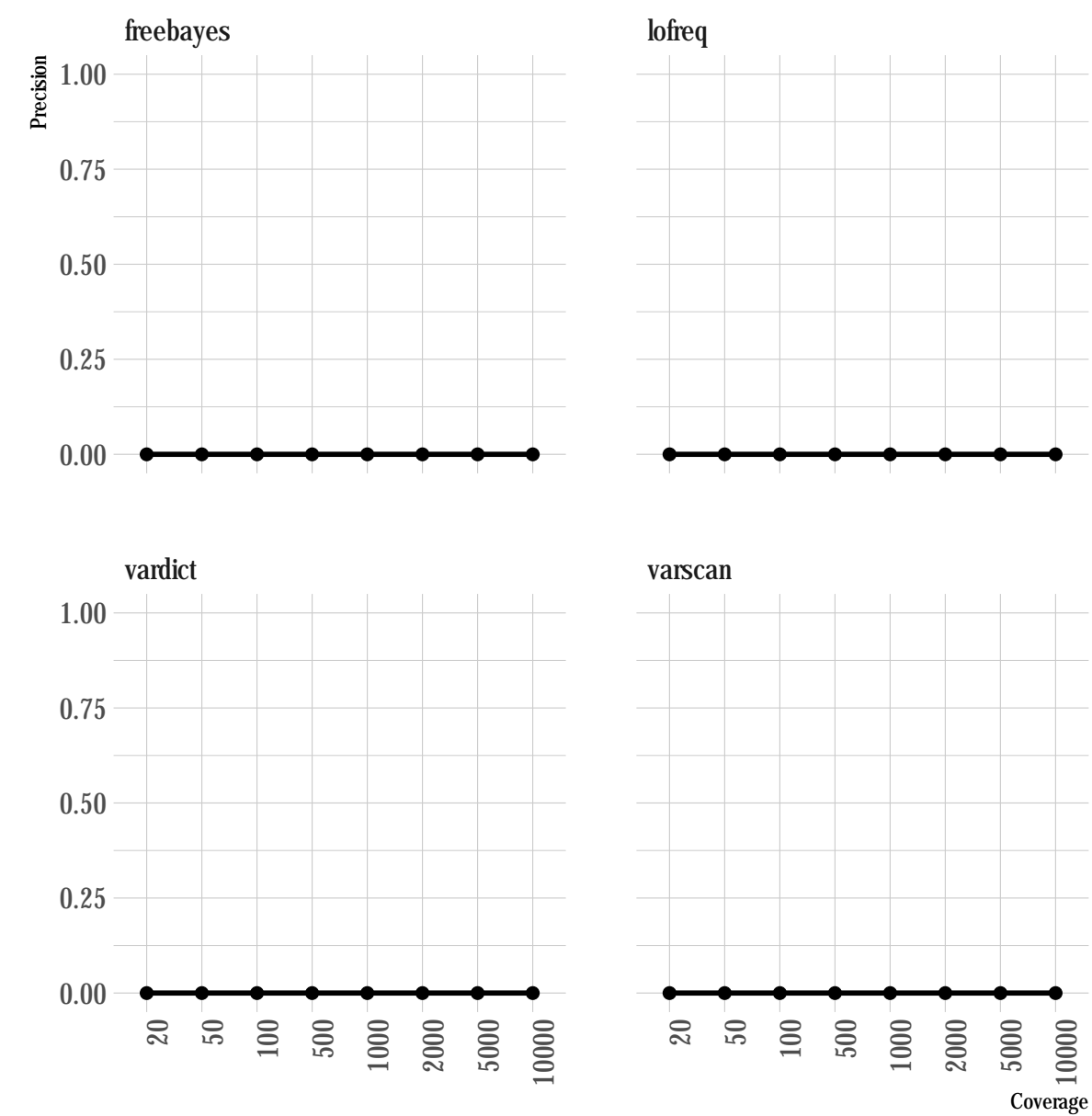

A

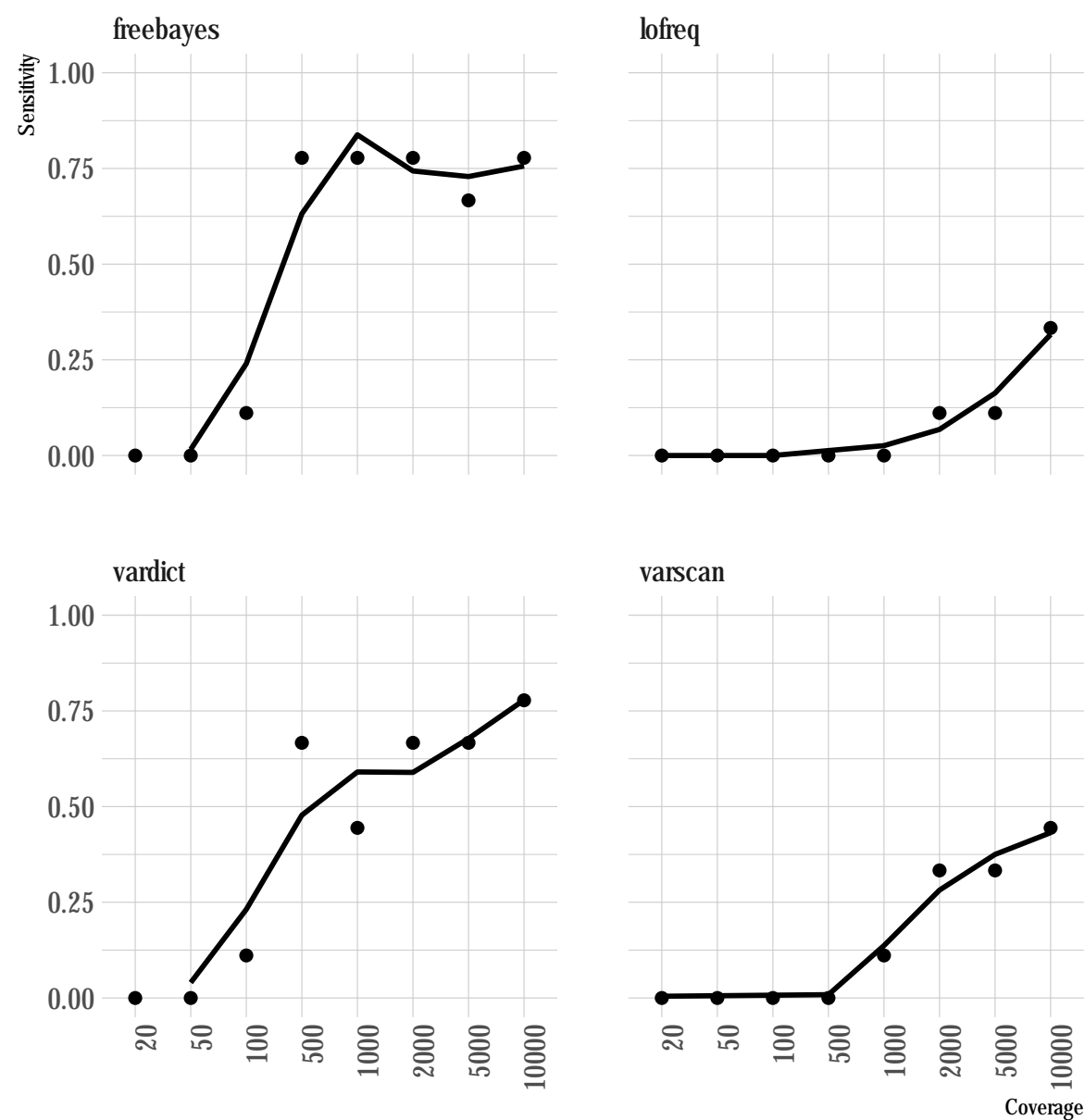

B

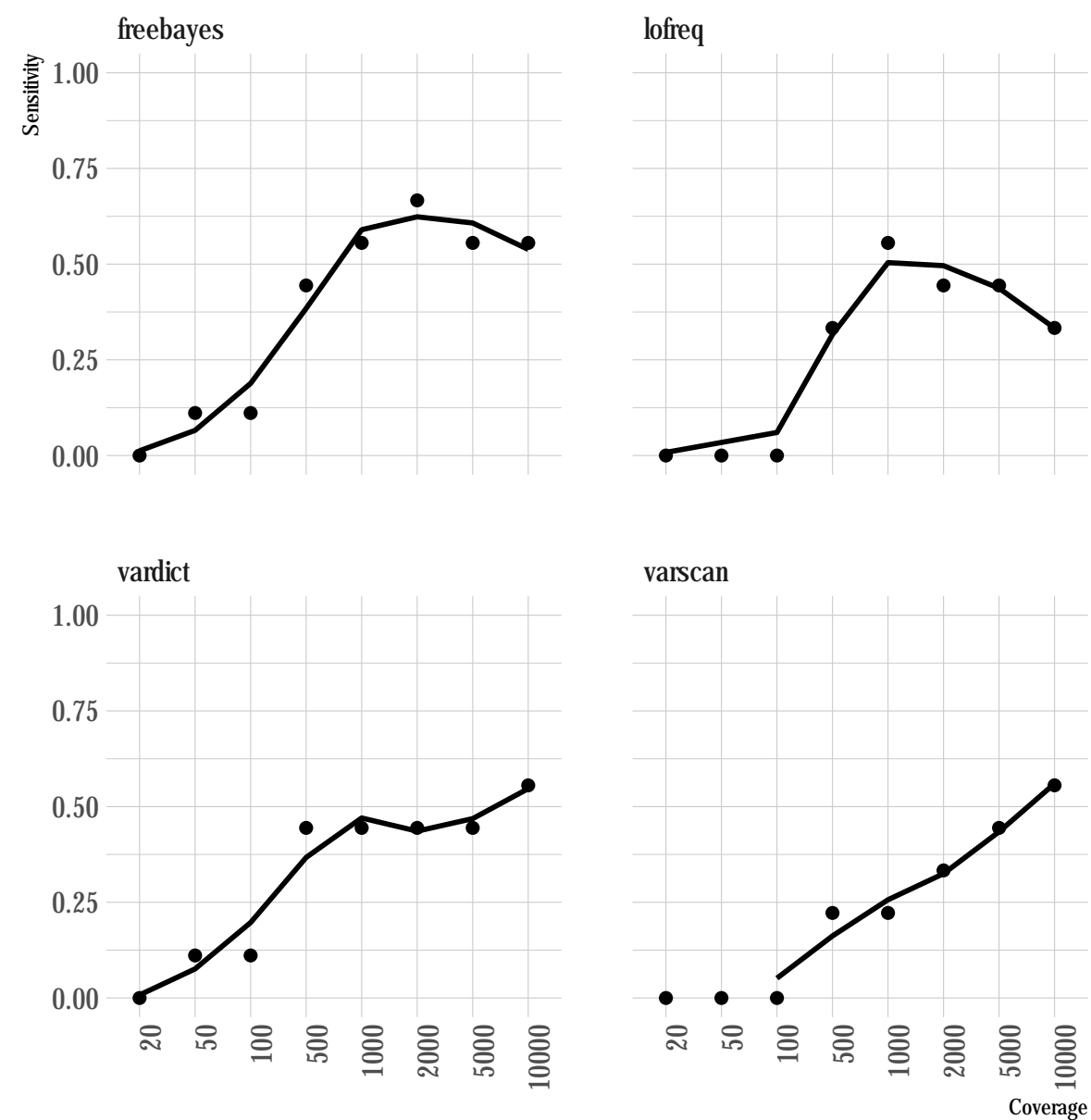

C

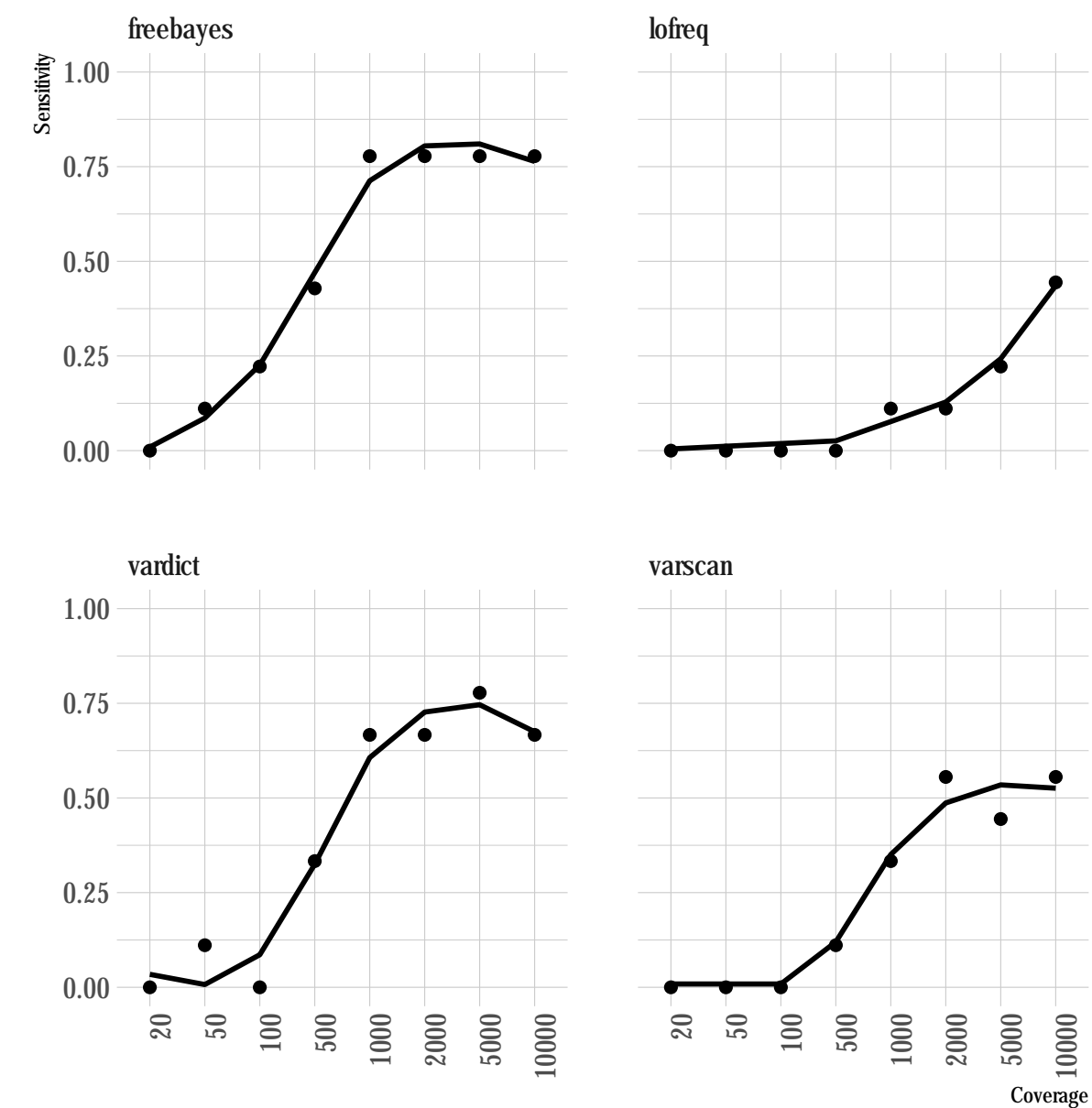

D

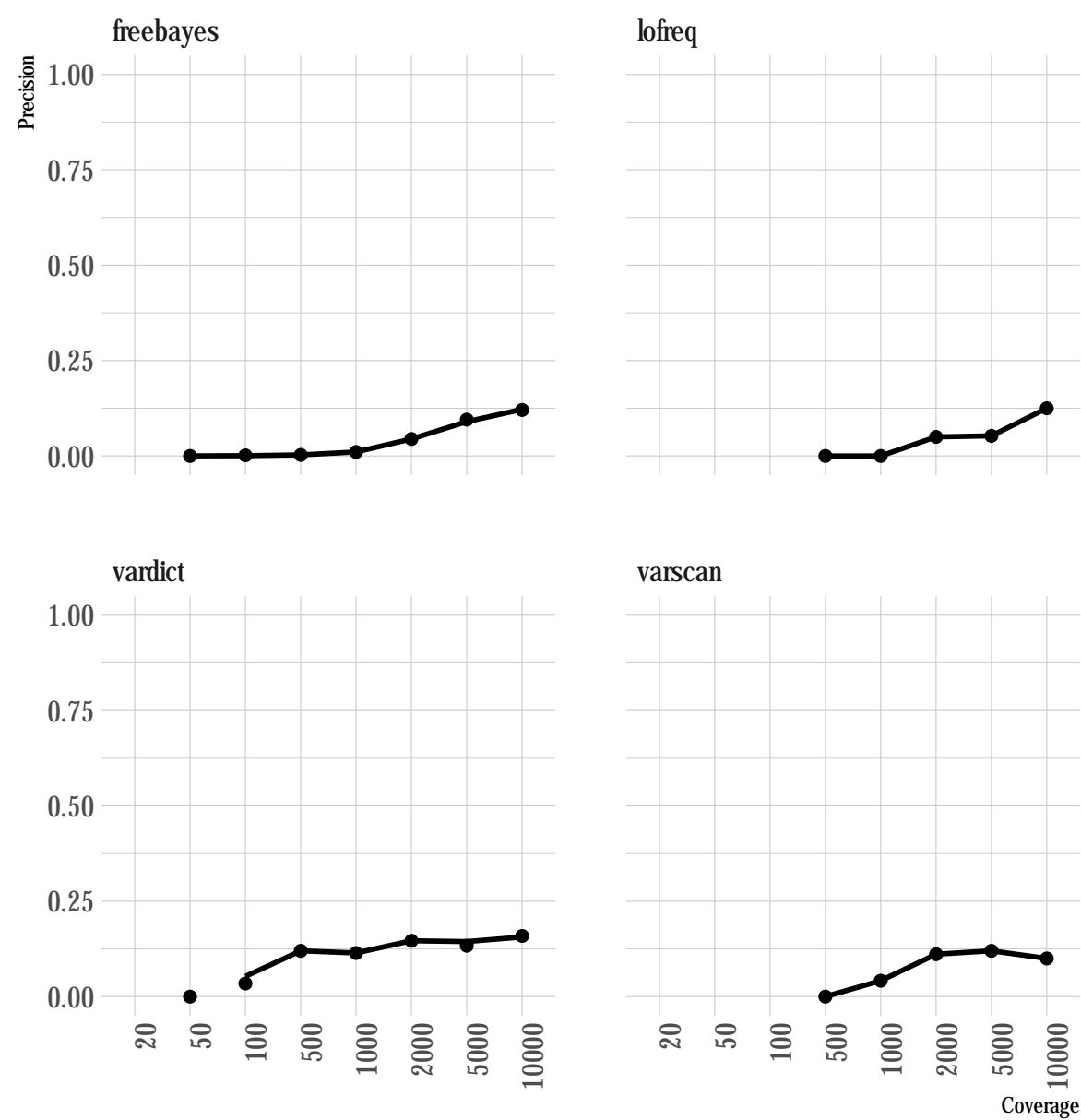

E

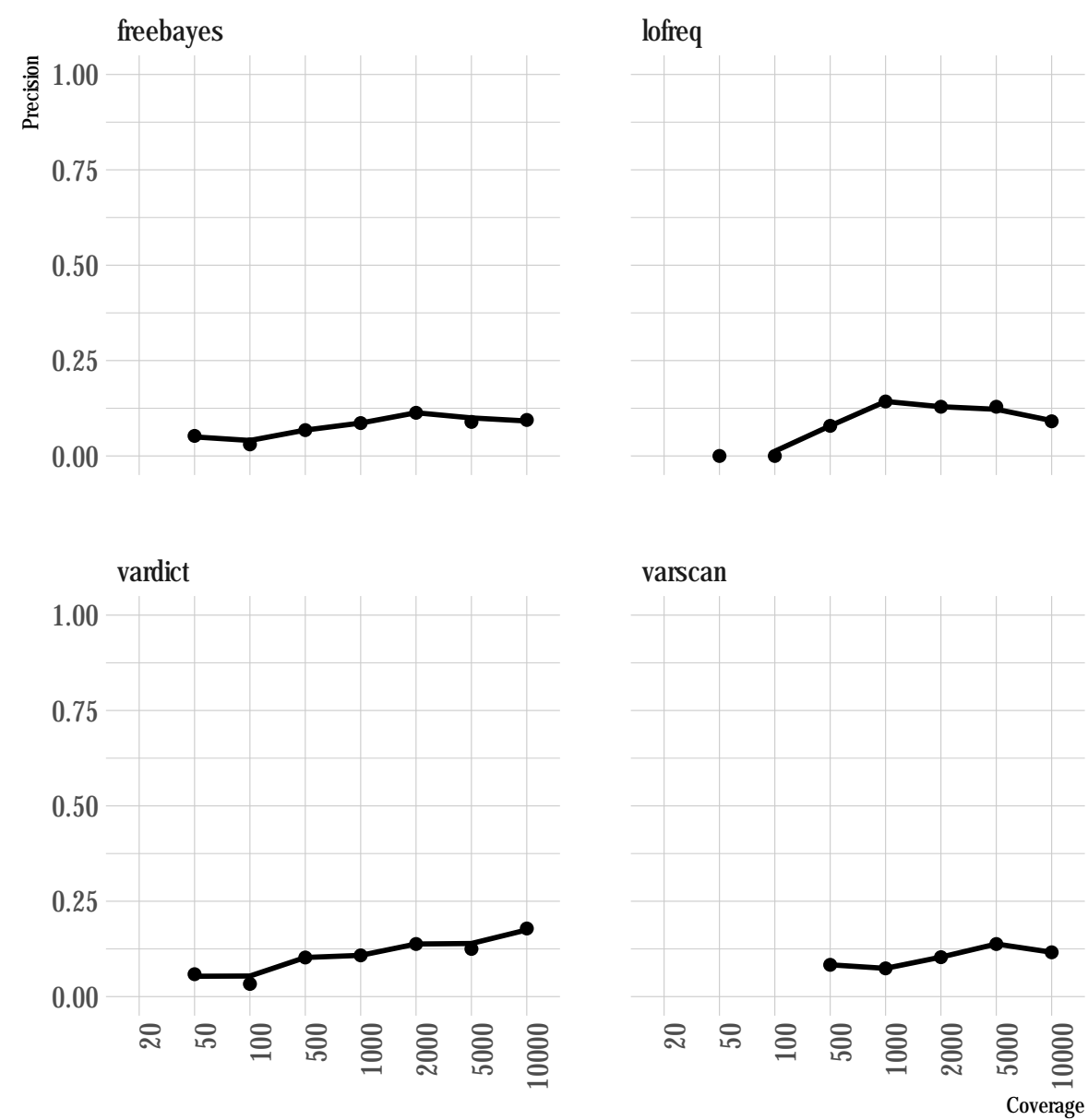

F

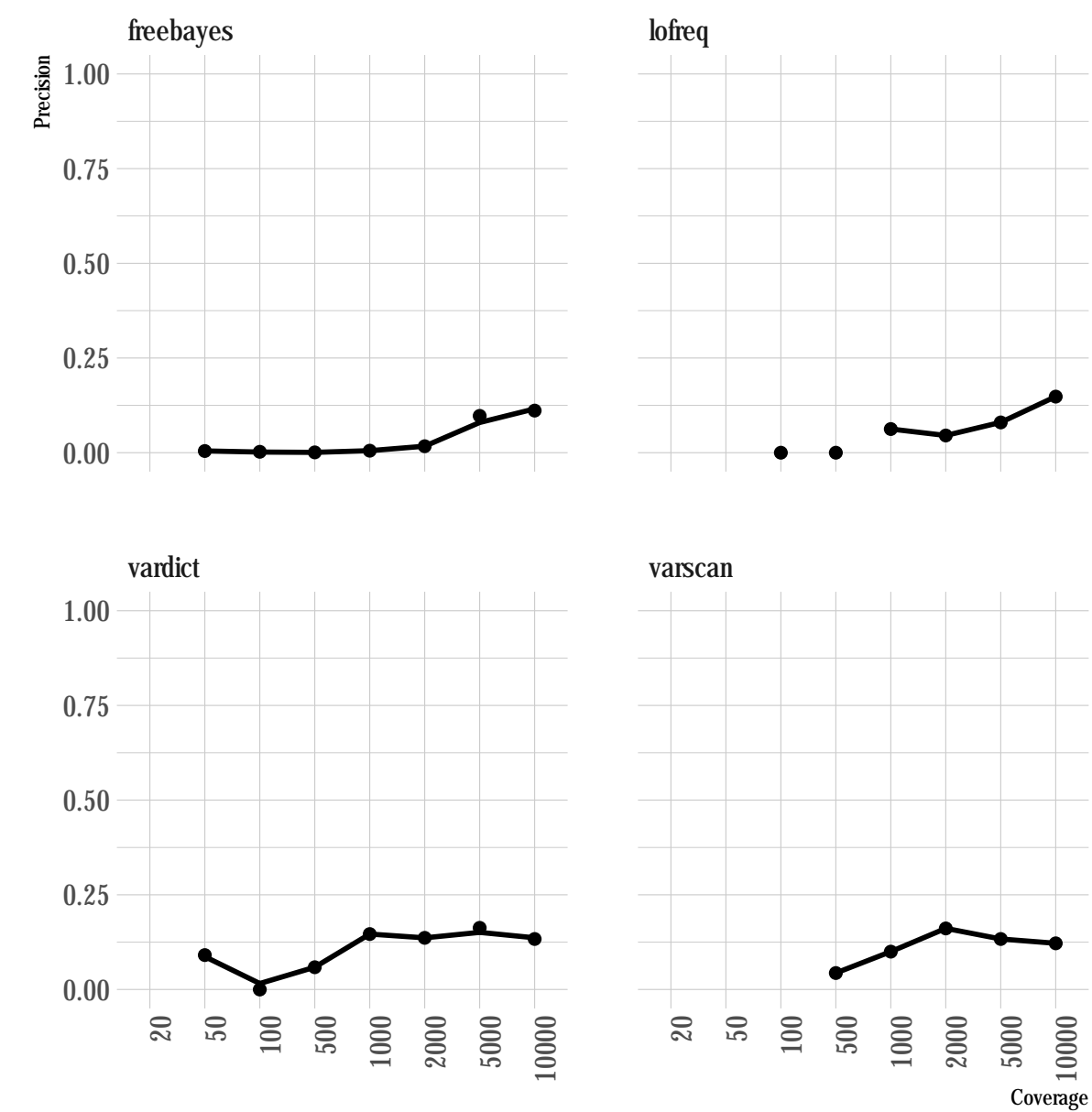

Supplement: Supplementary file 5 [file wellcomeopenres-3-16071-s0004.tgz › b9f525ab-3ec4-4500-8064-0e44c8ec49c7_supp_file_5.pdf]

A

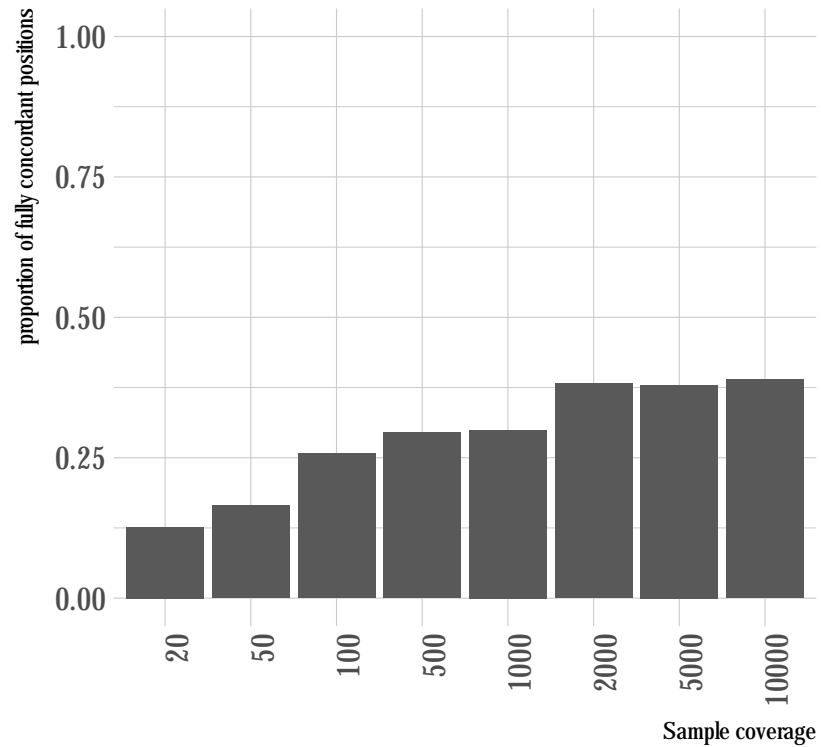

B

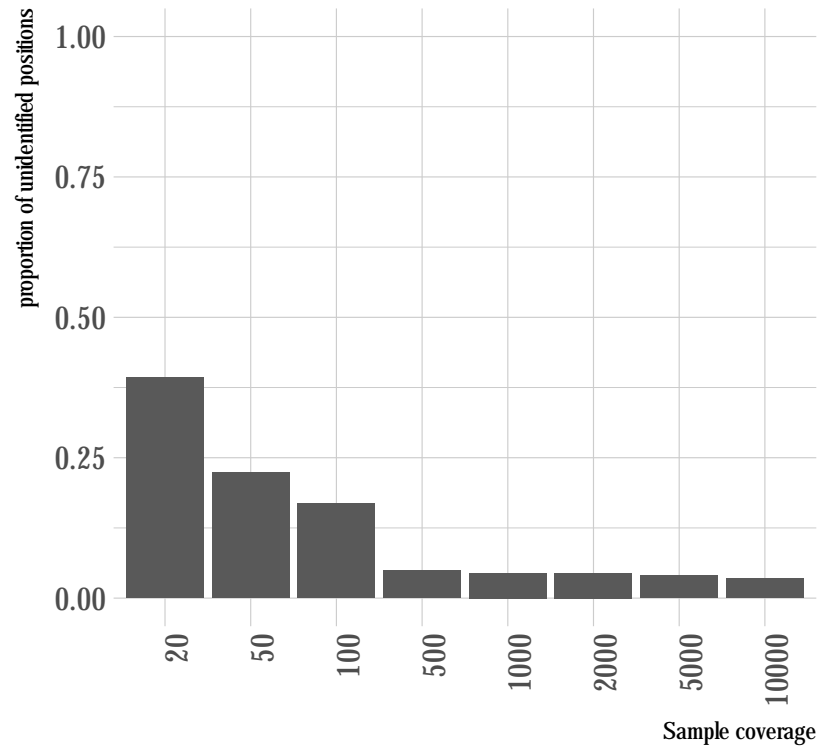

Supplement: Supplementary file 6 [file wellcomeopenres-3-16071-s0005.tgz › 486b10aa-00d3-412d-a238-bb2b8fb46190.pdf]

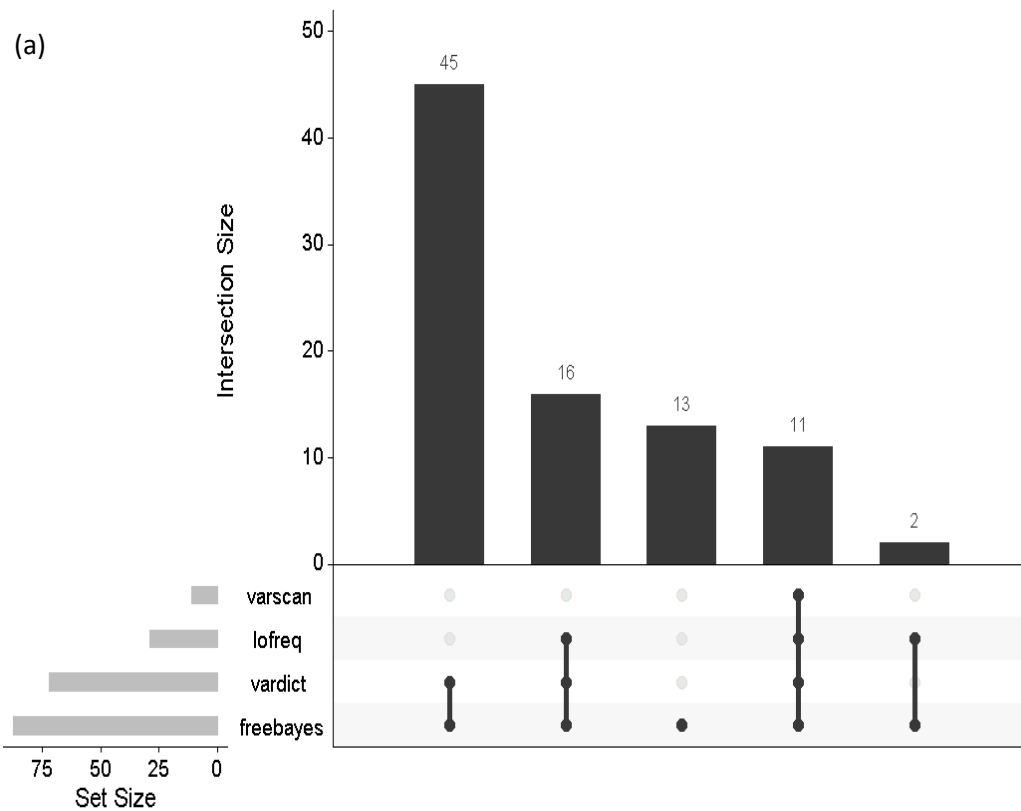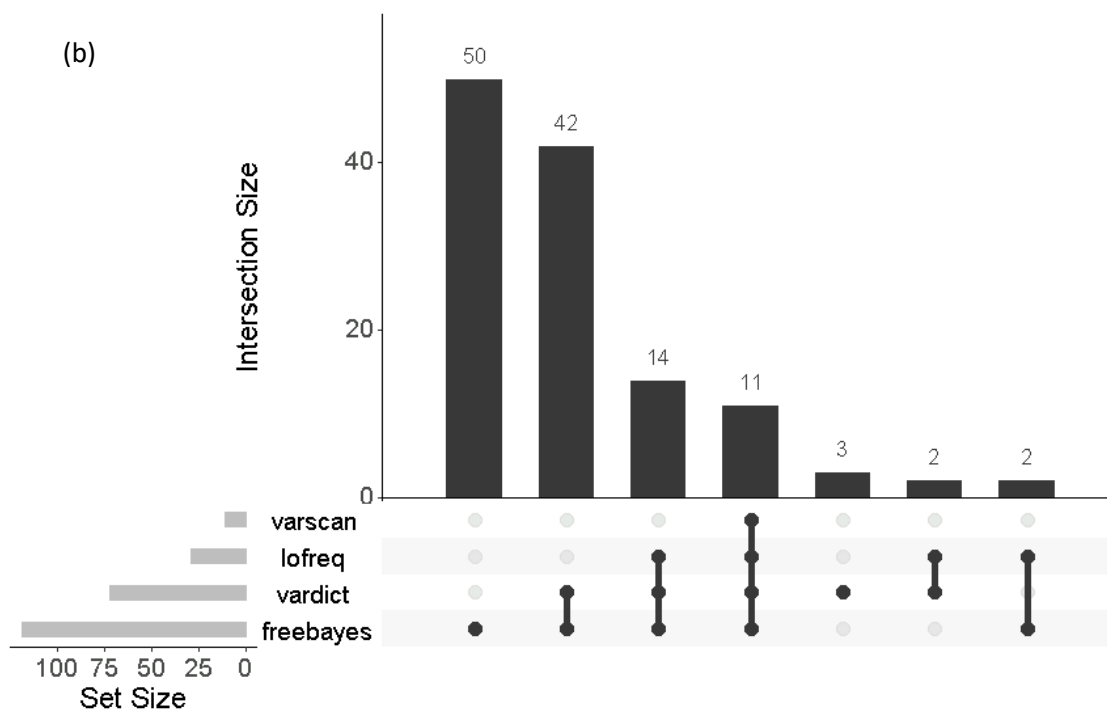

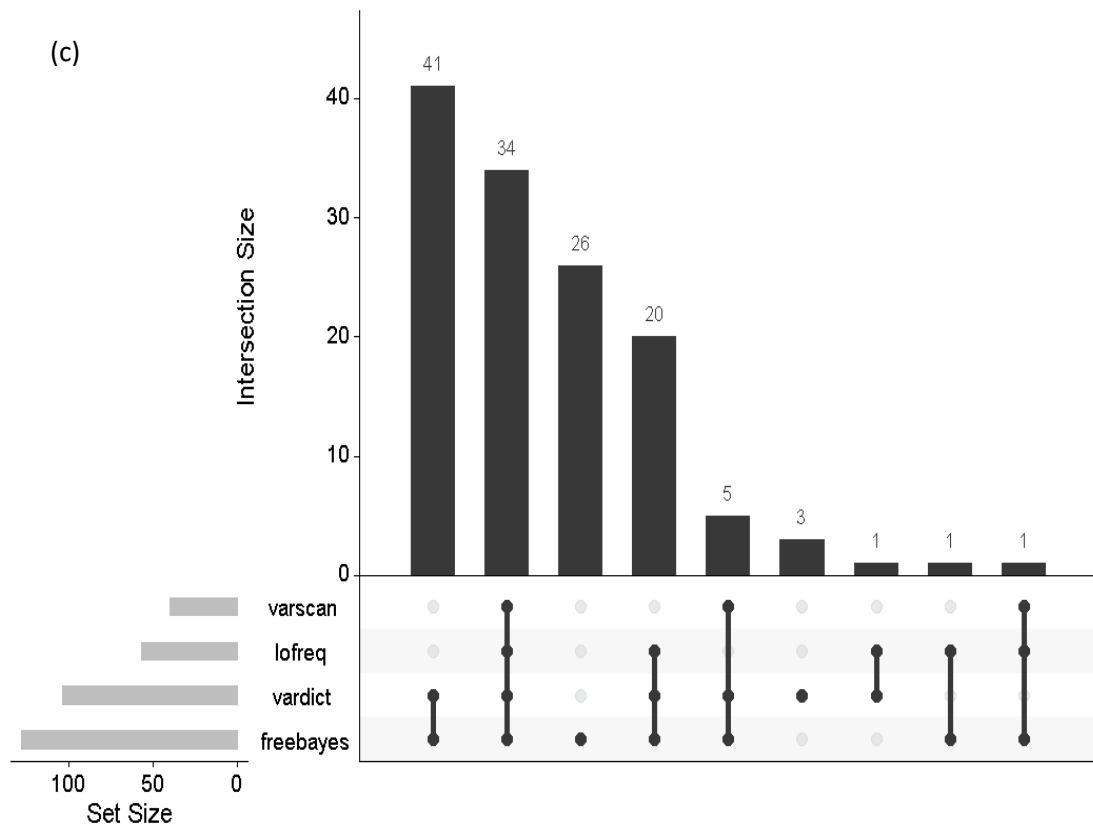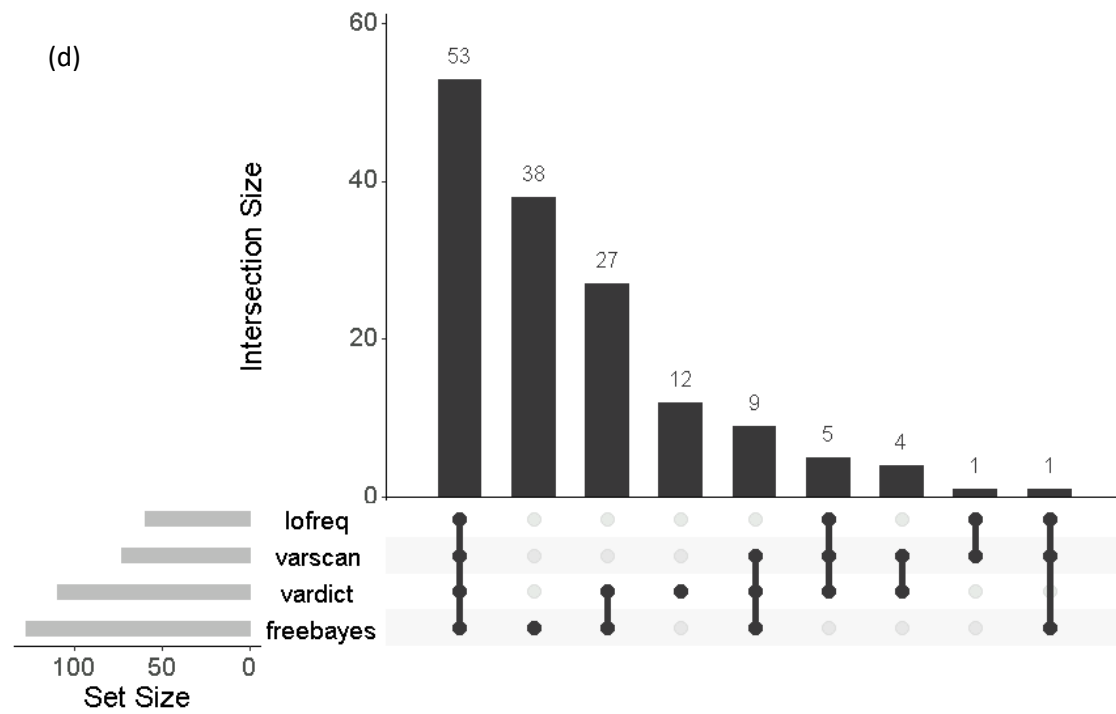



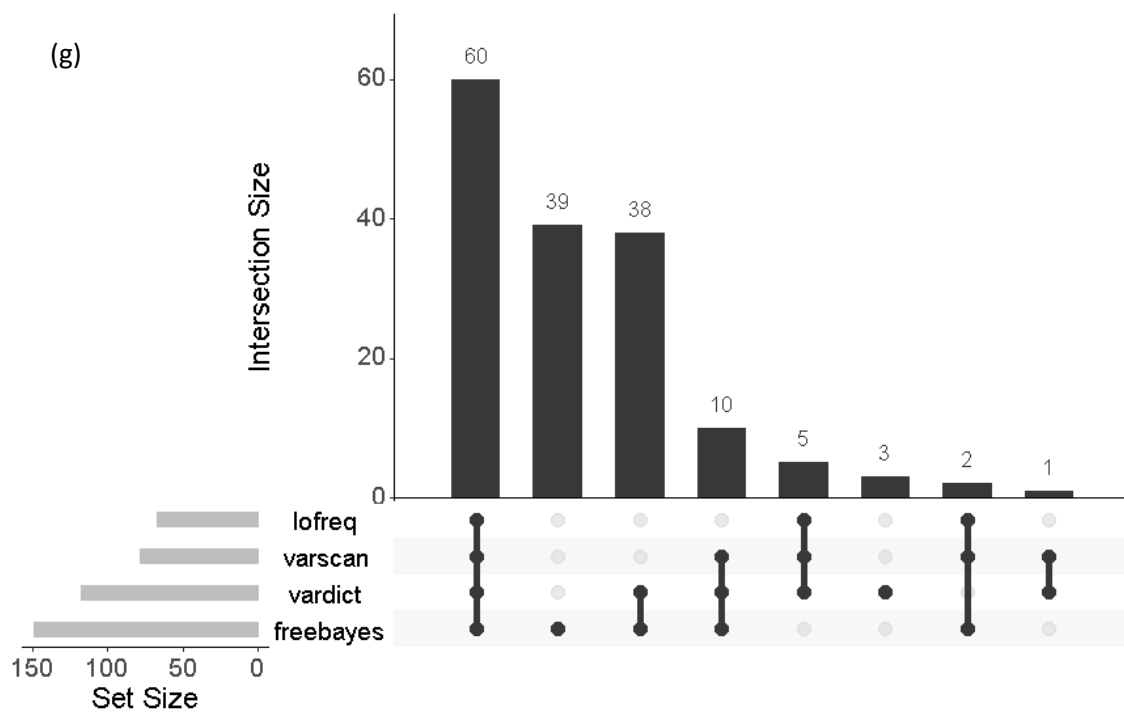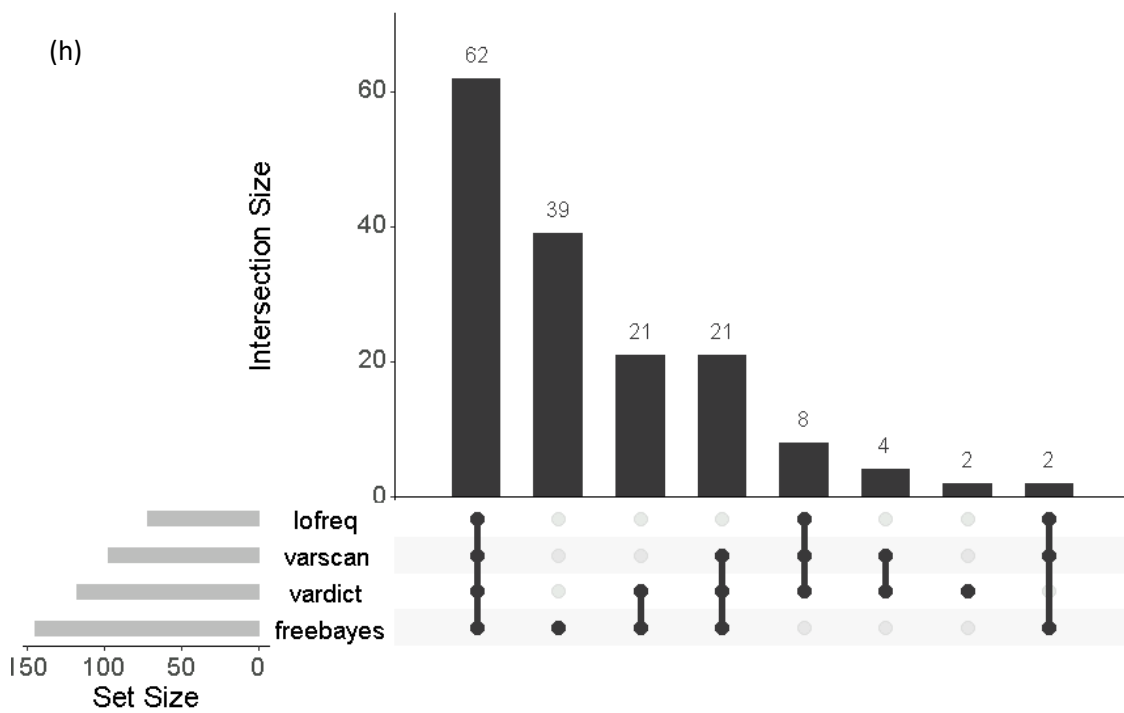

Supplement: Supplementary file 7 [file wellcomeopenres-3-16071-s0006.tgz › 0d489a9d-26a6-40f0-8882-f5b720c9f6ea.pdf]
