# Supplementary material for: Evaluating the performance of tools used to call minority variants from whole genome short-read data
Source: Wellcome Open Res. 2018 Sep 13;3:21. Originally published 2018 Mar 5. [Version 2] doi: 10.12688/wellcomeopenres.13538.2 (PMC6234735; doi:10.12688/wellcomeopenres.13538.2)
Supplement: Supplementary file 8 [file wellcomeopenres-3-16071-s0007.tgz › 0f455788-5903-413e-b918-f9e89213b303.docx]

**Supplementary Table 1:** 166 artificially generated nucleotide mutations with frequencies from 0-1

**Supplementary Table 2:** 156 artificially generated nucleotide mutations with frequencies below 0.5

**Supplementary Table 3:** **A breakdown of performance metrics of variant callers evaluated using the second dataset incorporated with an error profile derived from the set of reads used to assemble the reference genome.** The samples represent simulated dataset of varying average depth of coverage. True positive (TP), true negative (TN), false positive (FP) and false negatives (FN) were used to calculate performance metrics of each caller. FPR – False positive rate.

| Sample | Caller | TP | TN | FP | FN | Sensitivity | Specificity | Precision | FPR | Accuracy |
| --- | --- | --- | --- | --- | --- | --- | --- | --- | --- | --- |
| 1 (20X) | freebayes | 77 | 15039 | 0 | 89 | 0.4639 | 1 | 1 | 0 | 0.9941 |
|  | lofreq | 44 | 15039 | 0 | 122 | 0.2651 | 1 | 1 | 0 | 0.9920 |
|  | vardict | 77 | 15039 | 0 | 89 | 0.4639 | 1 | 1 | 0 | 0.9941 |
|  | varscan | 12 | 15039 | 0 | 154 | 0.0723 | 1 | 1 | 0 | 0.9899 |
| 2 (50X) | freebayes | 113 | 15039 | 0 | 53 | 0.6807 | 1 | 1 | 0 | 0.9965 |
|  | lofreq | 81 | 15039 | 0 | 85 | 0.4880 | 1 | 1 | 0 | 0.9944 |
|  | vardict | 110 | 15039 | 0 | 56 | 0.6627 | 1 | 1 | 0 | 0.9963 |
|  | varscan | 25 | 15039 | 0 | 141 | 0.1506 | 1 | 1 | 0 | 0.9907 |
| 3 (100X) | freebayes | 129 | 15039 | 0 | 37 | 0.7771 | 1 | 1 | 0 | 0.9976 |
|  | lofreq | 79 | 15039 | 0 | 87 | 0.4759 | 1 | 1 | 0 | 0.9943 |
|  | vardict | 105 | 15039 | 0 | 61 | 0.6325 | 1 | 1 | 0 | 0.9960 |
|  | varscan | 41 | 15039 | 0 | 125 | 0.2470 | 1 | 1 | 0 | 0.9918 |
| 4 (500X) | freebayes | 156 | 15033 | 6 | 10 | 0.9398 | 0.9996 | 0.9630 | 3.99E-04 | 0.9989 |
|  | lofreq | 82 | 15039 | 0 | 84 | 0.4940 | 1 | 1 | 0 | 0.9945 |
|  | vardict | 109 | 15038 | 1 | 57 | 0.6566 | 0.9999 | 0.9909 | 6.65E-05 | 0.9962 |
|  | varscan | 74 | 15039 | 0 | 92 | 0.4458 | 1 | 1 | 0 | 0.9939 |
| 5 (1000X) | freebayes | 157 | 15038 | 1 | 9 | 0.9458 | 0.9999 | 0.9937 | 6.65E-05 | 0.9993 |
|  | lofreq | 80 | 15039 | 0 | 86 | 0.4819 | 1 | 1 | 0 | 0.9943 |
|  | vardict | 105 | 15037 | 2 | 61 | 0.6325 | 0.9999 | 0.9813 | 1.33E-04 | 0.9959 |
|  | varscan | 78 | 15039 | 0 | 88 | 0.4699 | 1 | 1 | 0 | 0.9942 |
| 6 (2000X) | freebayes | 156 | 15039 | 0 | 10 | 0.9398 | 1 | 1 | 0 | 0.9993 |
|  | lofreq | 73 | 15037 | 2 | 93 | 0.4398 | 0.9999 | 0.9733 | 1.33E-04 | 0.9938 |
|  | vardict | 101 | 15039 | 0 | 65 | 0.6084 | 1 | 1 | 0 | 0.9957 |
|  | varscan | 84 | 15039 | 0 | 82 | 0.5060 | 1 | 1 | 0 | 0.9946 |
| 7 (5000X) | freebayes | 155 | 15039 | 0 | 11 | 0.9337 | 1 | 1 | 0 | 0.9993 |
|  | lofreq | 71 | 15035 | 4 | 95 | 0.4277 | 0.9997 | 0.9467 | 2.66E-04 | 0.9935 |
|  | vardict | 101 | 15039 | 0 | 65 | 0.6084 | 1 | 1 | 0 | 0.9957 |
|  | varscan | 81 | 15039 | 0 | 85 | 0.4880 | 1 | 1 | 0 | 0.9944 |
| 8 (10000X) | freebayes | 155 | 15039 | 0 | 11 | 0.9337 | 1 | 1 | 0 | 0.9993 |
|  | lofreq | 72 | 15033 | 6 | 94 | 0.4337 | 0.9996 | 0.9231 | 3.99E-04 | 0.9934 |
|  | vardict | 101 | 15039 | 0 | 65 | 0.6084 | 1 | 1 | 0 | 0.9957 |
|  | varscan | 98 | 15039 | 0 | 68 | 0.5904 | 1 | 1 | 0 | 0.9955 |

**Supplementary Table 4:** **A breakdown of performance metrics of variant callers evaluated using the third dataset generated with an error profile from a poorly sequenced sample.** The samples represent simulated dataset of varying average depth of coverage. True positive (TP), true negative (TN), false positive (FP) and false negatives (FN) were used to calculate performance metrics of each caller. FPR – False positive rate.

| Sample | Caller | TP | TN | FP | FN | Sensitivity | Specificity | Precision | FPR | Accuracy |
| --- | --- | --- | --- | --- | --- | --- | --- | --- | --- | --- |
| 1 (20X) | freebayes | 81 | 14995 | 44 | 85 | 0.4880 | 0.9971 | 0.6480 | 0.0029 | 0.9915 |
|  | lofreq | 28 | 15039 | 0 | 138 | 0.1687 | 1 | 1 | 0 | 0.9909 |
|  | vardict | 77 | 15039 | 0 | 89 | 0.4639 | 1 | 1 | 0 | 0.9941 |
|  | varscan | 9 | 15039 | 0 | 157 | 0.0542 | 1 | 1 | 0 | 0.9897 |
| 2 (50X) | freebayes | 118 | 14811 | 228 | 47 | 0.7152 | 0.9848 | 0.3410 | 0.0152 | 0.9819 |
|  | lofreq | 51 | 15039 | 0 | 115 | 0.3072 | 1 | 1 | 0 | 0.9924 |
|  | vardict | 107 | 15039 | 0 | 59 | 0.6446 | 1 | 1 | 0 | 0.9961 |
|  | varscan | 26 | 15039 | 0 | 140 | 0.1566 | 1 | 1 | 0 | 0.9908 |
| 3 (100X) | freebayes | 133 | 14237 | 802 | 32 | 0.8061 | 0.9467 | 0.1422 | 0.0533 | 0.9451 |
|  | lofreq | 56 | 15039 | 0 | 110 | 0.3373 | 1 | 1 | 0 | 0.9928 |
|  | vardict | 103 | 15038 | 1 | 63 | 0.6205 | 0.9999 | 0.9904 | 6.65E-05 | 0.9958 |
|  | varscan | 40 | 15039 | 0 | 126 | 0.2410 | 1 | 1 | 0 | 0.9917 |
| 4 (500X) | freebayes | 121 | 11395 | 3644 | 42 | 0.7423 | 0.7577 | 0.0321 | 0.2423 | 0.7575 |
|  | lofreq | 58 | 15039 | 0 | 108 | 0.3494 | 1 | 1 | 0 | 0.9929 |
|  | vardict | 116 | 15029 | 10 | 50 | 0.6988 | 0.9993 | 0.9206 | 6.65E-04 | 0.9961 |
|  | varscan | 71 | 15039 | 0 | 95 | 0.4277 | 1 | 1 | 0 | 0.9938 |
| 5 (1000X) | freebayes | 139 | 13754 | 1285 | 27 | 0.8373 | 0.9146 | 0.0976 | 0.0854 | 0.9137 |
|  | lofreq | 64 | 15039 | 0 | 102 | 0.3855 | 1 | 1 | 0 | 0.9933 |
|  | vardict | 115 | 15039 | 0 | 51 | 0.6928 | 1 | 1 | 0 | 0.9966 |
|  | varscan | 81 | 15039 | 0 | 85 | 0.4880 | 1 | 1 | 0 | 0.9944 |
| 6 (2000X) | freebayes | 140 | 14667 | 372 | 25 | 0.8485 | 0.9753 | 0.2734 | 0.0247 | 0.9739 |
|  | lofreq | 71 | 15039 | 0 | 95 | 0.4277 | 1 | 1 | 0 | 0.9938 |
|  | vardict | 116 | 15039 | 0 | 50 | 0.6988 | 1 | 1 | 0 | 0.9967 |
|  | varscan | 87 | 15039 | 0 | 79 | 0.5241 | 1 | 1 | 0 | 0.9948 |
| 7 (5000X) | freebayes | 142 | 15006 | 33 | 24 | 0.8554 | 0.9978 | 0.8114 | 0.0022 | 0.9963 |
|  | lofreq | 72 | 15039 | 0 | 94 | 0.4337 | 1 | 1 | 0 | 0.9938 |
|  | vardict | 116 | 15039 | 0 | 50 | 0.6988 | 1 | 1 | 0 | 0.9967 |
|  | varscan | 80 | 15039 | 0 | 86 | 0.4819 | 1 | 1 | 0 | 0.9943 |
| 8 (10000X) | freebayes | 145 | 15015 | 24 | 21 | 0.8735 | 0.9984 | 0.8580 | 0.0016 | 0.9970 |
|  | lofreq | 74 | 15039 | 0 | 92 | 0.4458 | 1 | 1 | 0 | 0.9939 |
|  | vardict | 121 | 15039 | 0 | 45 | 0.7289 | 1 | 1 | 0 | 0.9970 |
|  | varscan | 97 | 15039 | 0 | 69 | 0.5843 | 1 | 1 | 0 | 0.9955 |
