# Supplementary material for: Evaluating the performance of tools used to call minority variants from whole genome short-read data
Source: Wellcome Open Res. 2018 Sep 13;3:21. Originally published 2018 Mar 5. [Version 2] doi: 10.12688/wellcomeopenres.13538.2 (PMC6234735; doi:10.12688/wellcomeopenres.13538.2)
Supplement: Peer review history

# Peer review: Referee response for version 2

**DOI:** 10.21956/wellcomeopenres.16071.r33893

**Published:** 2018-11-13

**Authors:** Brad A. Chapman

**Affiliations:** 1 Department of Biostatistics, Harvard Public School of Health, Boston, MA, USA

Thank you for the detailed response and revision. The changes you've made greatly improve the paper and I especially appreciate the inclusion of run parameters for the tools and additional stratification plots.

 These revisions address all the previous suggestions, and my only additional comment from seeing Figure 5 and the run parameters was that most of these tools are producing a high percentage of low frequency noise which will result in poor precision when trying to identify variants in real samples. A useful followup would be explore filtering or ensemble methods to help improve this specificity.

 Thank you again for the work on this paper.

I have read this submission. I believe that I have an appropriate level of expertise to confirm that it is of an acceptable scientific standard.

# Peer review: Referee response for version 2

**DOI:** 10.21956/wellcomeopenres.16071.r33892

**Published:** 2018-10-02

**Authors:** Micha M. Bayer

**Affiliations:** 1 Information & Computational Sciences (ICS) group, James Hutton Institute, Dundee, UK

Thank you for addressing the issues raised in my review of Version 1. Everything has been resolved and I have no further comments to add.

I have read this submission. I believe that I have an appropriate level of expertise to confirm that it is of an acceptable scientific standard.

# Peer review: Referee response for version 1

**DOI:** 10.21956/wellcomeopenres.14703.r32418

**Published:** 2018-04-13

**Authors:** Micha M. Bayer

**Affiliations:** 1 Information & Computational Sciences (ICS) group, James Hutton Institute, Dundee, UK

This is a comparative review of variant calling tools with focus on the detection of minority variant calls, specifically with reference to virus data. The authors have provided data on four freely available tools that can be tuned to call minority variants, or are specifically designed for this purpose. The data used here was artificial reads generated with the ART-Illumina tool, which were spiked in with variants at known locations. This was replicated eight times at levels of read coverage spanning four orders of magnitude. The outcome of the experiment was a low level of concordance between tools. None of the tools were able to identify all of the spiked-in variants.

 The approach taken here is technically sound, as it is based on a strictly defined truth set, although I am slightly uncomfortable with the lack of real data (but equally I am aware of the methodological problems real data presents in this kind of scenario). The study is useful for the wider community as a) it reiterates the point that using a single tool for a given bioinformatics task comes with the risk of missing information and b) it provides concrete pointers as to which tools perform well in this scenario.

 My main reservation is that there is no mention of the importance of parameter settings. Different parameter values can affect variant calling outcomes dramatically, even when just comparing different runs of the same tool. FreeBayes alone has over 70 command line parameters, many of these continuous variables. This makes for an incredibly large parameter space, and using different sets of parameter values can potentially have much more of an impact on the results than the choice of tool. This should at least be mentioned in the Discussion section.

 There also doesn’t seem to be any data on how the individual tools were parameterised in this study. Were the defaults used in each case? Presumably not, given that we are looking for minority variants (and tools like FreeBayes appear to be developed and parameterised with a human germline use case in mind). This information is critical for reproducibility and must be included (even if it is just a statement saying the defaults were used).

 My other comments below reference the online PDF version of the article.

**Abstract**

- Results section: change “FreeBayes identified majority” to “FreeBayes identified the majority”
- Conclusions section: change “impact” to “impacts”

 

**Introduction**

- para 1 line 7: is the “and” redundant?
- para 5 line 7: comma after “callers”

 

**Methods**

- para 1 line 1: “we considered eleven published,open-source tools” I counted six excluded tools in Table 1, plus four that have been taken through to evaluation = 10
- para 2 (“Artificial datasets”): The concept of the error profile has not been well explained. This section needs to be expanded. I would like to see some description of what the error profile consists of, how it has been derived from the FASTQC data, and how it has been applied to the reads in practice.
- para 3 line 2:“at varying frequencies” – what were they? I presume we are talking about the proportion of reads at a spiked-in variant site that carried the alternate allele at this site. This information strikes me as absolutely critical for this paper – this is about minority variants after all. Please provide this data.
- para 4 line 8: “written as a comma separated (CSV) files” should be “written as a comma separated (CSV) file”

 

**Results**

- para 3 line 1: “FreeBayes identified majority” should be “FreeBayes identified the majority”
- para 3 line 7: “it missed majority” should be “it missed the majority”
- General comment: Vardict should get a mention in this section, as it actually had better recall rates than FreeBayes in 6 out of 8 samples.

 

**Discussion**

- para 1 line 5: change “ascertain” to “distinguish”
- para 3 line 1: “Sensitivity for majority” should be “Sensitivity for the majority”

 

**Table 3**

- As far as I can tell, the counts for FN + TP should always add up to 166. This is not the case for the majority of the Freebayes runs (except for Freebayes with samples 1 and 3, which are ok).

 

**Supplementary File S1**

- To ensure reproducibility, please include the BAMSurgeon command line statement for spiking in the variants.

 

**Supplementary Figure 2**

- It is not apparent what the individual figures/pages in the PDF file represent. Please label these appropriately.

I have read this submission. I believe that I have an appropriate level of expertise to confirm that it is of an acceptable scientific standard, however I have significant reservations, as outlined above.

# Response

**Published:** 2018-08-13

**Authors:** George Githinji

**Affiliations:** KEMRI-Wellcome Trust Research Programme, Kenya

We are grateful for taking time to review this work and for providing useful comments that we have considered in this new draft of the manuscript.

- We agree on the challenges of obtaining and using the appropriate actual data when conducting these types of analysis. We have done our best to generate reliable artificial datasets based on an actual RSV reference genome and error profiles from two sequenced samples using the methods described in this manuscript.
- In regard to the importance of parameter settings and lack of data on how individual tools were parameterized, we have explicitly stated the parameter settings in the variant calling section and in the Supplementary File 4. In the discussion section, we highlight the challenge of exploring all possible parameter settings.
- The concept of the error profile has is now well explained in paragraph 1 of the artificial datasets section in the current manuscript.
- The actual frequencies used to spike the variants are now described and provided in Supplementary file 5.
- There was a bug in the script used to calculate the FN and TP counts in FreeBayes runs. This has been corrected and updated to reflect the true FN and TP counts. A revised table and subsequent figures have been provided.

# Peer review: Referee response for version 1

**DOI:** 10.21956/wellcomeopenres.14703.r31459

**Published:** 2018-03-22

**Authors:** Brad A. Chapman

**Affiliations:** 1 Department of Biostatistics, Harvard Public School of Health, Boston, MA, USA

The authors validate methods to call low frequency minority variants from viral samples. Detection of minority species in viral population sequencing helps improve treatments by matching drugs to the full spectrum of sub-species, and allows tracking of sub-species within outbreaks.

 The paper uses synthetic datasets generated by the ART read simulator (https://www.niehs.nih.gov/research/resources/software/biostatistics/art/) with randomly generated variants. While this won't fully describe the complexity of a real viral mixture, it does provide a solid baseline for assessing callers. The authors used mutation error profiles from good and bad sequencing samples to model different error rates, and evaluated the impact of differing coverage on sensitivity and specificity.

 My main suggestion is to stratify comparisons by variant frequency to assess how callers do with different low frequency events, and I provide more specific comments below.

**Methods suggestions**

- The stratifications by coverage, error rates and callers are great for identifying the effect of different callers on detection. The one missing component is the allele frequency of the generated variants. The paper does not define the range of frequencies generated, and this will have a large impact on the ability of detection across callers. Looking through the list of generated variants (Githinji_2018_variant.list.tab) the range varies from high frequency (90%+) to very low frequency (<1%). Since the focus is on low frequency detection, I'd suggest stratifying the results into bins: standard (>25%), moderately low frequency (5-25%) and low frequency (<5%) and examine the caller and depth detection within these bins. Pending the results of this, the authors may want to generate additional low frequency variants to help differentiate caller methods.
- A confusing aspect of evaluating the depth metrics is that the number of possible true positives differs between sample depths. In Table 3, the FreeBayes TP + FNs are 166, 165 and 166 for 20x, 50x and 100x. However they change to 61, 55 and 55 for 500x, 1000x and 200x. Other callers seem to be consistent. Why does FreeBayes have different total variant numbers across depth?

**Paper suggestions**

- Table 1 should get compressed into a single paragraph saying that HaploptypeCaller, Platypus and mpileup target germline calling and not low frequency detection. It doesn't need to be a separate table.
- Table 2 should be replaced into a link to descriptions of true/false positives/negatives and does not need to be a separate figure in the paper.
- The title should reflect that this paper describes viral calling (versus, say low frequency somatic variant detection).

I have read this submission. I believe that I have an appropriate level of expertise to confirm that it is of an acceptable scientific standard, however I have significant reservations, as outlined above.

# Response

**Published:** 2018-08-13

**Authors:** George Githinji

**Affiliations:** KEMRI-Wellcome Trust Research Programme, Kenya

We are very grateful for taking time to review this work and for providing useful comments which we have considered in the new version of the manuscript:

- All the paper suggestions have been considered table 1 and table 2 were removed and replaced with text descriptions.
- We have added a new figure comparing the allele frequency of the spiked and called variants. We highlight the observations in the result and discussion sections. In addition, we provided a stratified result of the variants frequency and discuss how the callers perform for different low frequency events. We also provided an addition set of spiked variants at frequency below 50%.
- The results section in table 3 have been revised to reflect the correct number of TP and FN for FreeBayes. Figure was also revised to reflect the new changes.
